# ﻿Taxonomic studies on *Sanicula* L. (Apiaceae) from China (V): Revision of the *Sanicula serrata* H. Wolff complex: morphological delimitation from *S. potaninii* Bobrov, synonymization of allied taxa, and lectotypifications

**DOI:** 10.3897/phytokeys.265.169937

**Published:** 2025-10-21

**Authors:** Hui-Min Li, Wei Zhou, Bao-Cheng Wu, Xu-Dong Ma, Jun Wen, Chun-Feng Song

**Affiliations:** 1 Jiangsu Key Laboratory for Conservation and Utilization of Plant Resources, Institute of Botany, Jiangsu Province and Chinese Academy of Sciences (Nanjing Botanical Garden Mem. Sun Yat-Sen), Nanjing 210014, Jiangsu, China Jiangsu Key Laboratory for Conservation and Utilization of Plant Resources, Institute of Botany, Jiangsu Province and Chinese Academy of Sciences (Nanjing Botanical Garden Mem. Sun Yat-Sen) Nanjing China

**Keywords:** Carrot family, herbaria, morphology, Umbelliferae

## Abstract

Based on observations of living plants in the field and examination of herbarium specimens (including type materials), we demonstrate that Sanicula serrata var. serrata (Apiaceae) differs from *S. potaninii* by having single fruit bearing squamose spines – occasionally tuberculate-spiculate or apically prickly – whereas *S. potaninii* usually bears three fruits with densely uncinate spines. This morphological distinction is further supported by differences in their biogeographic distributions. In addition, *S. tienmuensis*, *S. elongata*, S. tienmuensis var. pauciflora, and *S. langaoensis* are found to be conspecific with *S. serrata*, while S. serrata var. uncinata is conspecific with *S. potaninii*; we therefore reduce these taxa to synonymy accordingly. Lectotypifications are proposed for *S. elongata* and S. tienmuensis var. pauciflora.

## ﻿Introduction

**Figure 1.**
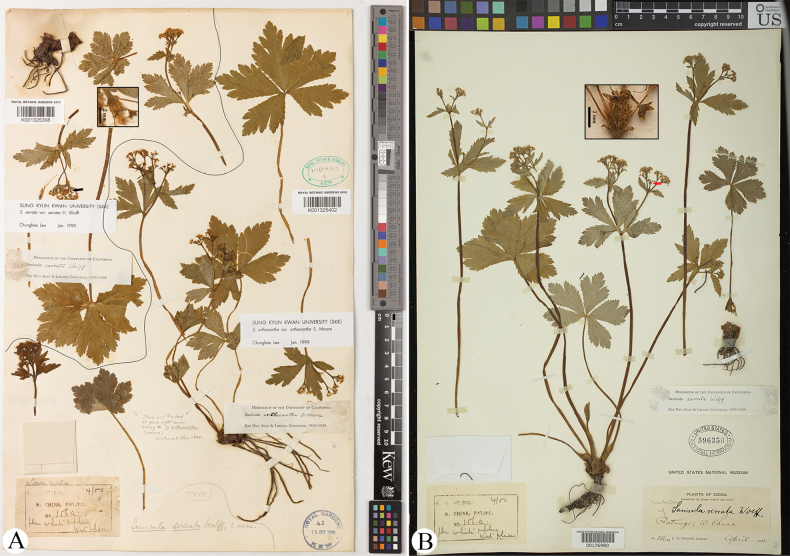
Lectotype (upper left; A) and isolectotype (B) sheets of Sanicula serrata var. serrata. Arrows indicate fruits with squamose spines.

*Sanicula serrata* H. Wolff was originally described based solely on the collection *E.H. Wilson 156A* (K001325398, NY00406258-right lower part, US00126980; but only the B sheet was cited in the protologue; Fig. 1, only sheets K001325398 and US00126980 shown here) from western Hubei. Wolff (1913) characterized the species by its leaf segments with entire margins irregularly and sub-duplicate serrate and the teeth minutely apiculate at the apex. Its staminate flowers are 4–6 per umbellule, borne on pedicels ca. 3 mm long. The dried petals were described as pale yellow, suborbicular in shape, abruptly constricted at the base, and bearing an inflexed ligule of equal length (2–3 times narrower than the lamina, ca. 2 mm long). The anthers were noted to be violet-green, while the styles were capillary. Immature fruits bore straight, serrulate, or sparsely and shortly uncinate spines.

Since its description, *S. serrata* has been recognized by subsequent authorities including Handel-Mazzetti (1933), Shan (1936), Hiroe (1979), Liou (1979), Fu (1981), Wu (1984), Wu (1993), Liu (1997), Sheh and Phillippe (2005), and Pimenov (2017). Its distribution encompassed western Hubei, eastern Qinghai, Sichuan, southeastern Xizang (Tibet), and northwestern Yunnan (Sheh and Phillippe 2005; Pimenov 2017).

**Figure 2.**
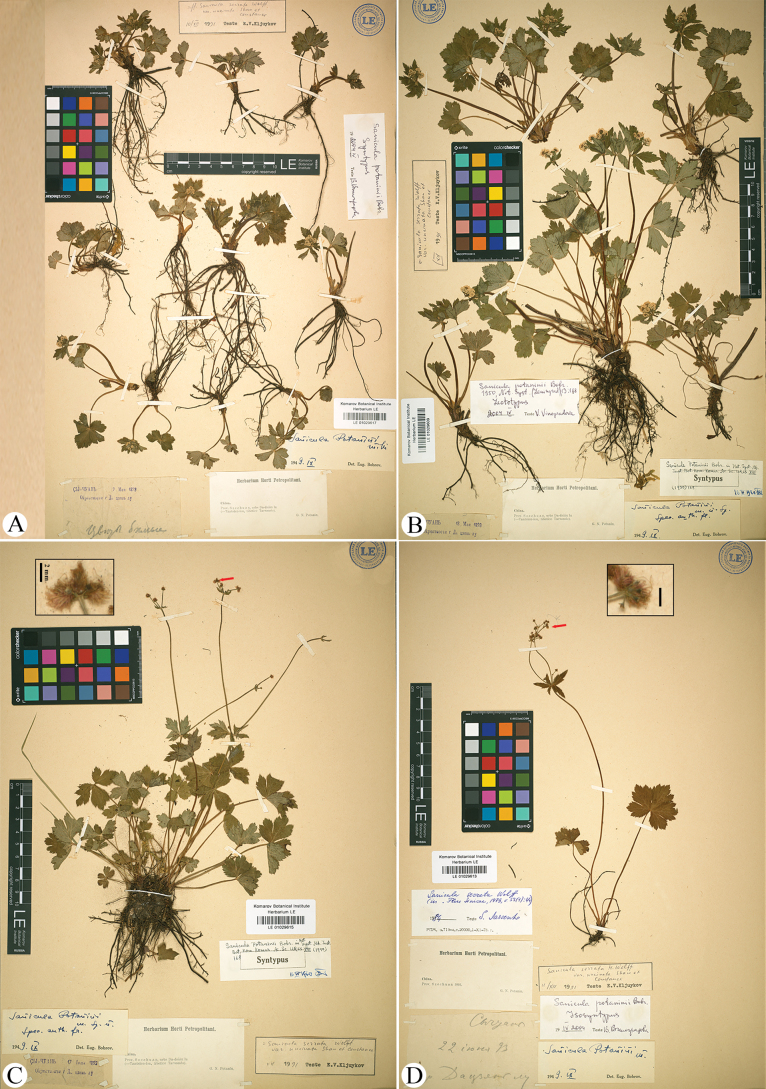
Syntype sheets of *Sanicula potaninii*. All specimens represent original material cited by Bobrov (1950); specimen B (LE01029609) is designated by Vinogradova (2010) as the lectotype. Arrows in B, C. indicate densely uncinate spines.

*Sanicula potaninii* Bobrov was described based on five gatherings collected by G.N. Potanin (Bobrov 1950): specimens from 9 May 1893 (LE01029617; Fig. 2A), 18 May 1893 (LE01029608–LE01029612, PE00025857; Fig. 2B, sheet LE01029609 shown here), 27 May 1893 (LE01029616), 17 June 1893 (LE01029615, PE00025858; Fig. 2C, only sheet LE01029615 shown here), and 22 June 1893 (LE01029613, LE01029614; Fig. 2D, only sheet LE01029613 shown here), from Kangding [K’ang-ting, Tatsienlu, Dartsedo, Da-dsian-lu, Tantsien-lou or Tarsando], western Sichuan. In the protologue, the author explicitly referred to multiple specimens as types according to the International Code of Nomenclature (Art. 9.5; Turland et al. 2025), these are regarded as syntypes. In the original description, the author emphasized the presence of basal leaves with distinctly crenate-serrate margins, the teeth being attenuate and mucronate. Staminate flowers (5–8 per umbellule) were pedicellate and arranged radially, while fertile flowers (2–3 per umbellule) were sessile and centrally clustered. The petals were white, the calyx teeth reflexed, and the styles significantly exserted. The fruits were ovoid, 3–4 mm in length, with a purple-tinged, squamulose ventral surface and a dorsal surface bearing uncinate purple spines. Bobrov (1950) distinguished this species from the ca. 11 gerontogeous taxa of S. sect. Eusanicula Wolff (= S. sect Sanicla DC.; Wolff 1913) by its broadly membranous-vaginate pedicels at the base, conspicuously crenate-serrate leaf blades, and fruits with squamulose ventral surfaces and uncinate dorsal spines. Subsequently, Vinogradova (2010) recognized *S. potaninii* as a distinct species, whereas Sheh and Phillippe (2005) reduced it to synonymy under *S. astrantiifolia* H. Wolff & Kretschmer, and Pimenov (2017) treated it as a synonym of *S. serrata*, all without providing justification.

**Figure 3.**
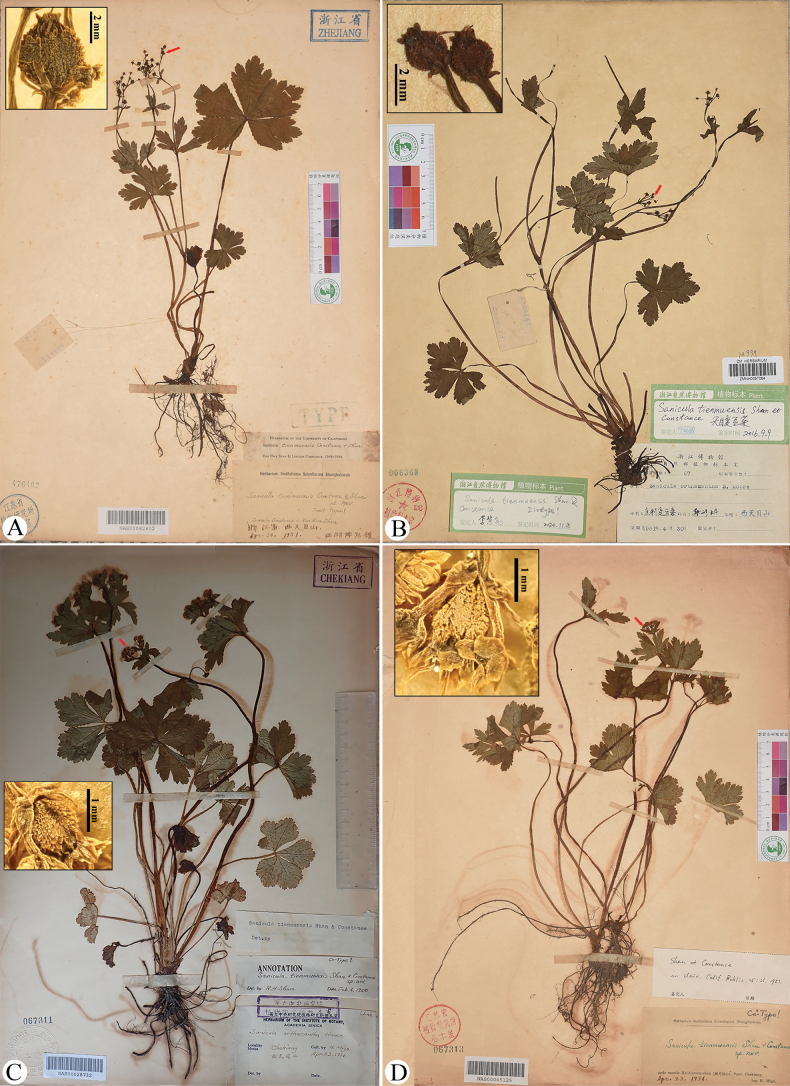
Holotype (A), isotype (B), paratype (C), and a duplicate of the paratype (D) sheets of Sanicula tienmuensis var. tienmuensis. Arrows indicate fruits with squamose spines.

*Sanicula tienmuensis* Shan & Constance was described from two samplings from West Tianmushan [T’ien mu shan], Lin’an, Hangzhou, Zhejiang, China. These included *West Lake Museum 67* (ZMNH0057084, ZMNH0057085-part, NAS00082802; Fig. 3A, B, the sheets NAS00082802 and ZMNH0057084 shown here) and *H. Migo s.n.* (NAS00028732, NAS00082803, NAS00045125–NAS00045127; Fig. 3C, D, the sheets NAS00028732, NAS00045125 shown here). The NAS sheet (Fig. 3C) of the first collection was designated as the holotype. In the protologue, the authors compared this species to *S. tuberculata* Maxim., *S. serrata*, and *S. orthacantha* S. Moore, and stated that it differed from *S. tuberculata* by having a solitary (vs. usually three) fertile flower, fewer (vs. ca. 20) staminate flowers, branched (vs. usually unbranched) stems and inflorescences, and fruits with squamous tubercles lacking prickles (vs. tuberculate fruits with short dorsal uncinate prickles). Although resembling *S. serrata* and *S. orthacantha* in general habit, foliage, and solitary fertile flowers, *S. tienmuensis* was distinguished by its urceolate-globose fruits and subsessile staminate flowers (Shan and Constance 1951). Subsequent authorities have recognized this species (Hiroe 1979; Liou 1979; Sheh and Phillippe 2005; Pimenov 2017), with its distribution encompassing southwestern Sichuan and Zhejiang (Sheh and Phillippe 2005; Pimenov 2017).

**Figure 4.**
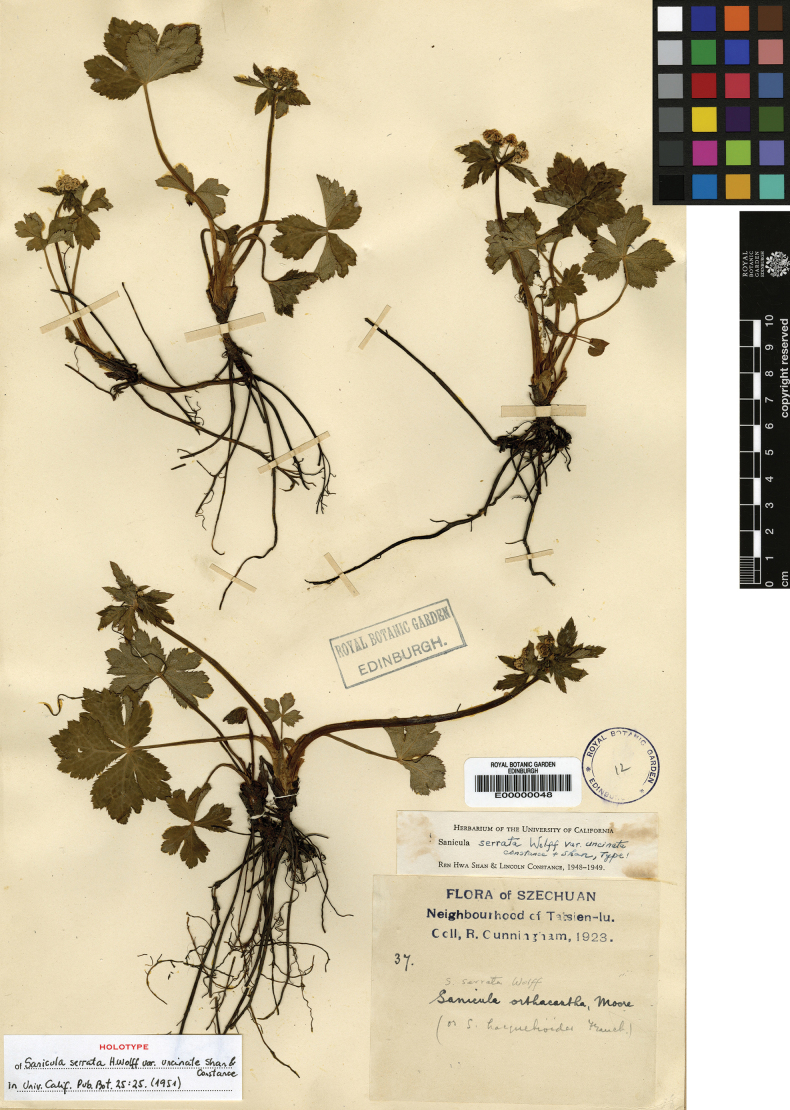
Holotype sheet of Sanicula serrata var. uncinata.

Sanicula serrata var. uncinata Shan & Constance was characterized based on a single collection, *Cunningham 37* (E00000048; Fig. 4), from the Cheto Valley, Kangding, western Sichuan. In the protologue, the authors noted that this variety consistently differed from the type variety, i.e., S. serrata var. serrata, by its uncinate (vs. short, curved spicules above, squamose below) ovaries and typically three (vs. solitary) fertile flowers (Shan and Constance 1951). Following its description, this variety was recognized only by Pimenov (2017), who noted its endemic distribution in southwestern Sichuan.

**Figure 5.**
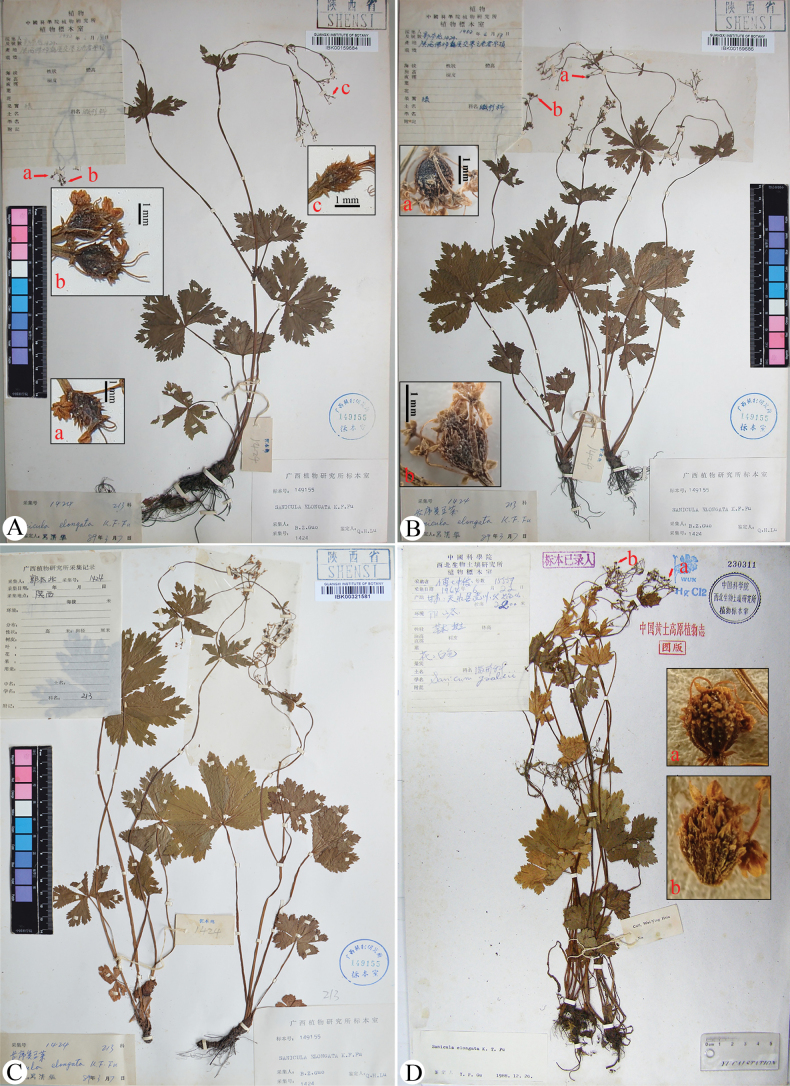
Lectotype (A), isolectotypes (B, C), and paratype (D) sheets of *Sanicula elongata*. Arrows indicate fruits covered with densely squamose spines, sometimes ending in minute prickles.

*Sanicula elongata* K.T. Fu was described based on two collections: one was *P.C. Kuo 1424* (IBK00159684–IBK00159686, KUN0465506; Fig. 5A–C, only three sheets shown here), collected from Laojunling (Lau-chun-ling), Mei County, Shaanxi [Shan-his, Shensi]; the other was *K.T. Fu 15559* (WUK230311; Fig. 5D), collected from Dangchuan Township (referred to as Tang-chuan), Tianshui, Gansu [Kansu]. In the protologue, Fu (1979 in Liou 1979) cited the former collection as the type but did not designate a specific specimen as the holotype. He posited that this species was closely related to S. serrata var. serrata, yet it could be differentiated by its tightly clustered, spiny fruits, which lacked a hook, and by its more upright, flexible stems, particularly noted for their high flowering productivity. The species was subsequently recognized by Fu (1981), Sheh and Phillippe (2005), and Pimenov (2017), with its distribution documented in Gansu and Shaanxi provinces (Sheh and Phillippe 2005).

**Figure 6.**
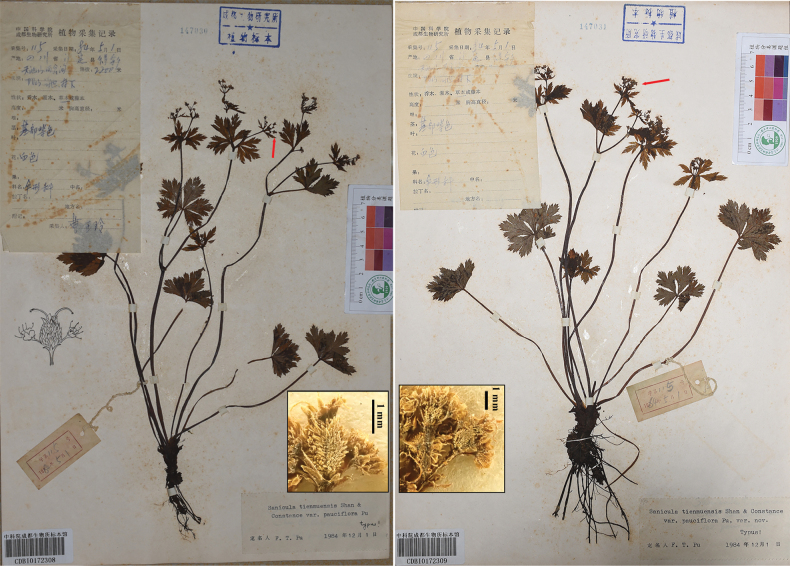
Lectotype (A) and isolectotype (B) sheets of Sanicula serrata var. pauciflora.

Sanicula tienmuensis var. pauciflora Shan & Pu was described based on one collection *Y.L. Cao 115* (CDBI0172308, CDBI0172309; Fig. 6; the collector was not *Y.J. Li* as stated in the protologue), from Mount Tianchi, Desui Town, Luding [Lu ting] County, Sichuan. In the protologue, the authors (1989) did not designate a type for S. tienmuensis var. pauciflora, and distinguished this variety from the type variety, S. tienmuensis var. tienmuensis, by its bearing of only 2–3 (vs. 5–6) staminate flowers per umbellule. Following its description, this variety was recognized by Wu (1993), Sheh and Phillippe (2005), and Pimenov (2017). Recently, Song et al. (2024) elevated this taxon to species rank, naming it *S. pauciflora* (R.H. Shan & F.T. Pu) B.N. Song & X.J. He.

**Figure 7.**
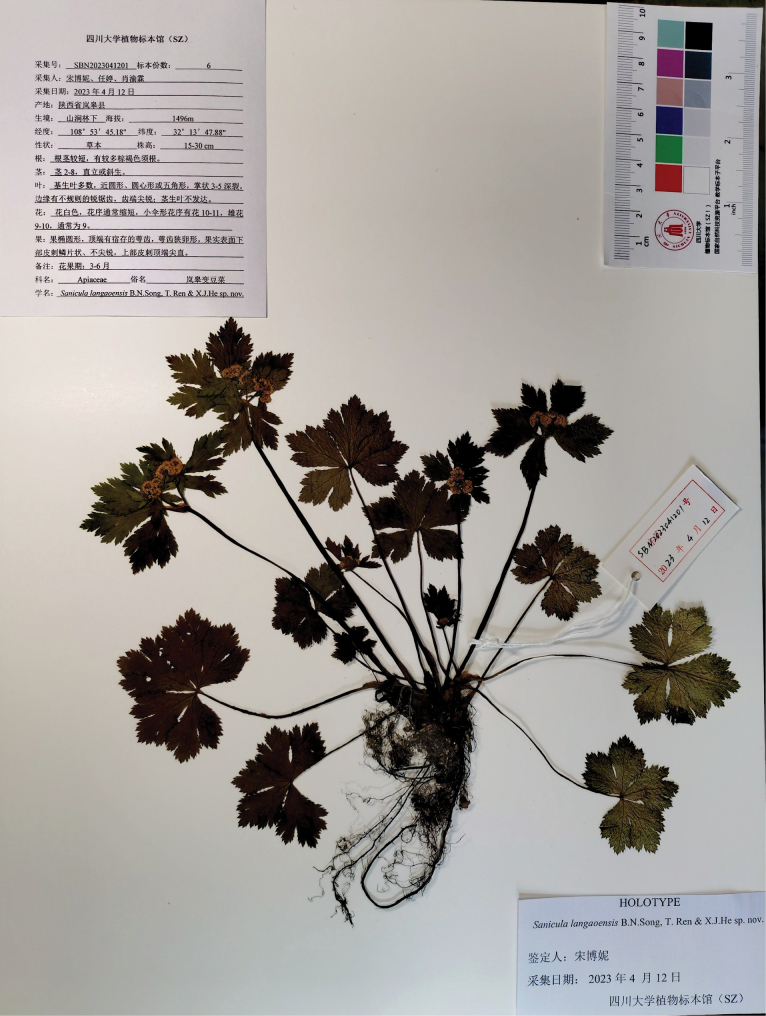
Holotype sheet of *Sanicula langaoensis*.

*Sanicula langaoensis* B.N. Song, T. Ren & X.J. He was described based on one gathering, *B.N. Song, T. Ren & X.J. He SBN2023041201* (SZ; Fig. 7), from Lan’gao County, Shaanxi [Shan-his, Shensi] (not “Shanxi” as stated in the protologue). In the protologue, the authors (2024) stated that the leaf blade was subrounded, round-cordate, or pentagonal, palmately 3–5-parted, with sharply and irregularly serrate margins; 9–10 staminate flowers per umbellule (usually 9); 1 fertile flower per umbellule; calyx teeth were narrowly ovate, ca. 1 mm long; fruits were ellipsoid, with the proximal end bearing scalariform prickles (not acute), with the distal end bearing acute prickles.

Through comprehensive examination of abundant herbarium specimens (including type material) and field observations, we found that the taxonomic delimitation among Sanicula serrata var. serrata, *S. potaninii*, S. tienmuensis var. tienmuensis, S. serrata var. uncinata, *S. elongata*, and S. tienmuensis var. pauciflora has long been problematic. Persistent confusion among authorities has primarily resulted from insufficient morphological characterization, as reflected in the works of Handel-Mazzetti (1933), Shan (1936), Shan and Constance (1951), Hiroe (1979), Liou (1979), Fu (1981), Wu (1984), Liu (1997), Wu (1993), Sheh and Phillippe (2005), and Pimenov (2017). The aim of our study is to elucidate the diagnostic morphological characteristics of Sanicula serrata var. serrata, delineate its taxonomic distinction from *S. potaninii*, and reassess the taxonomic identity of S. serrata var. uncinata, S. tienmuensis var. tienmuensis, *S. elongata*, S. tienmuensis var. pauciflora, and *S. langaoensis.* We also propose lectotypifications for *S. elongata* and S. tienmuensis var. pauciflora.

## ﻿Material and methods

For morphological comparisons, we conducted a thorough examination of specimens or high-resolution images of related *Sanicula* L. from the following herbaria: BM, CDBI, E, GZTM, HBG, HIB, HITBC, HNWP, HTC, HZ, IBK, K, KUN, KUZ, L, LBG, LE, NAS, NY, O, P, PE, PEY, SM, SWFC, SZ, US, WUK, XBGH, and ZM. Field observations were conducted across 17 populations in Anhui, Hubei, Sichuan, Shaanxi, Yunnan and Zhejiang Provinces (Table 1). Of these, five populations are highlighted: one from Shennongxi, Badong [Pa tung] County, Hubei, the type locality of S. serrata var. serrata; one from Mount Paoma in Kangding County, western Sichuan, the type locality of *S. potaninii* and S. serrata var. uncinata; one from West Tianmushan, Lin’an, Hangzhou, Zhejiang, the type locality of S. tienmuensis var. tienmuensis; one from Mount Zhongyan, Weibin district, Baoji City, north-western Shaanxi, neighboring Mei County, the type locality of *S. elongata*; one from Mount Tianchi, Desui Town, Luding County, Sichuan, the type locality of S. tienmuensis var. pauciflora. The morphological comparisons presented are the result of a comprehensive analysis of both herbarium specimens and fresh materials collected during our fieldwork.

**Table 1.** The field collection information of 15 populations of *Sanicula serrata* and two populations of *S. potaninii*.

Taxon	Voucher	Locality	Note	Figure
*Sanicula serrata*	*H.M. Li, Y.S. Zhang & Y. Xu 1110* (NAS)	Anhui, Huoshan County, Cangping Village		
	*Y.S. Zhang LHM1111* (NAS)	Hubei, Badong County, Shennongxi	the type locality of S. serrata var. serrata	Figs 8, 10A, 11A
	*F. Tan LHM1700* (NAS)	Hubei, Wufeng County, Xiaolong Village		
	*F. Tan LHM1701* (NAS)	Hubei, Nanzhang County, Xuping Town		
	*H.M. Li & L. Zhao 1114* (NAS)	Zhejiang, Anji, Mount Longwang		
	*H.M. Li & L. Zhao 1116* (NAS)	Zhejiang, Hangzhou, Lin’an, west Tianmushan	the type locality of S. tienmuensis var. tienmuensis	Figs 10B, 11B, 12
	*H.M. Li & L. Zhao 1117* (NAS)	Zhejiang, Jinhua City, Pan’an County, Mount Dapan		
	*H.M. Li & C.F. Song 1207* (NAS)	Shaanxi, Baoji City, Mount Zhongyan	neighboring the type locality of *S. elongata*	Figs 10C, 11C, 14
	*H.M. Li & C.F. Song 1219* (NAS)	Shaanxi, Liuba County		
	*H.M. Li & C.F. Song 1223* (NAS)	Shaanxi, Lueyang County		
	*H.M. Li & C.F. Song 1231* (NAS)	Shaanxi, Ankang City, Zhenping County		
	*H.M. Li & C.F. Song 1232* (NAS)	Shaanxi, Shangluo City, Zhen’an County		
	*H.M. Li & C.F. Song 1233* (NAS)	Shaanxi, Ankang City, Ningshaan County		
	*H.M. Li & Y.S. Zhang 1167* (NAS)	Sichuan, Luding, Desui Town, Mount Tianchi	the type locality of Sanicula tienmuensis var. pauciflora	Figs 10D, 11D, 17
	*B.C. Wu, H.M. Li & J.W. Zhu 130* (NAS)	Yunnan, Gongshan		
*Sanicula potaninii*	*H.M. Li & Y.S. Zhang 1163* (NAS)	Sichuan, Kangding, Mount Paoma	the type locality of *S. potaninii* and S. serrata var. uncinata	Figs 9, 10E
	*H.M. Li & C.F. Song 1200* (NAS)	Shaanxi, Mei County, Mount Taibai		

Numerical analyses of inflorescence length, from the first visible umbel to the terminal umbel on each individual inflorescence, were conducted based on measurements from herbarium specimens of Sanicula serrata var. serrata collected from nine populations in Hubei, Zhejiang, Shaanxi, and Sichuan (Suppl. material 1). ImageJ (Abràmoff et al. 2004) was used to obtain quantitative measurements and visualizations were generated in R (de Micheaux et al. 2013).

## ﻿Results and discussion

**Figure 8.**
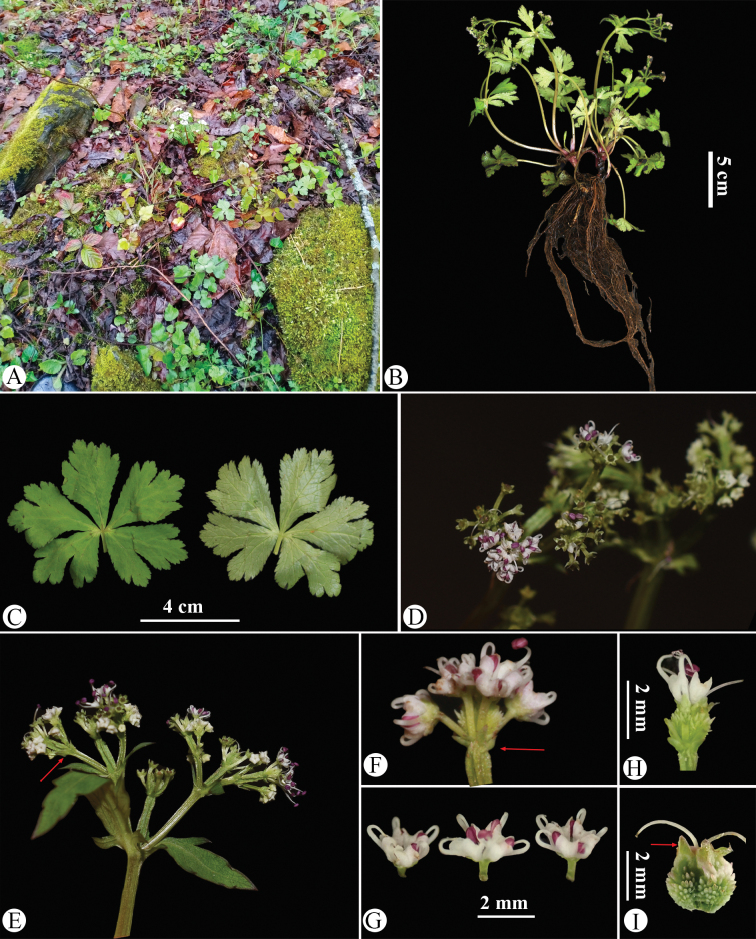
Sanicula serrata var. serrata in the wild (China, Hubei, Badong County, Shennongxi, the type locality of S. serrata var. serrata). A. Habitat; B. Habit; C. Leaf (left: adaxial surface; right: abaxial surface); D. Portion of the inflorescence (top view); E. Portion of the inflorescence (side view), arrow indicating the involucral bract; F. Umbellule (side view), arrow indicating the involucellate bracteoles; G. Staminate flower (side view); H. Fertile flower with immature fruit; I. Mericarps, arrow indicating the calyx teeth. Photographed by Hui-Min Li.

Our examination of the type material of Sanicula serrata var. serrata (Fig. 1; Table 2) reveals basal leaf segments with irregularly crenulate-serrate and subduplicately serrate margins throughout. The umbellules typically contain approximately six flowers, comprising five staminate and one fertile flower. The calyx teeth are broadly ovate and shortly acuminate. The fruits bear densely squamose spines, occasionally terminating in minute recurved projections, but are distinctly non-hooked. These morphological characteristics were further corroborated by field observations of living specimens (Fig. 8; Table 2) from Shennongxi, Badong County, Hubei — the type locality of S. serrata var. serrata.

**Table 2.** Diagnostic characters based on examinations of the type material and populations from the type locality, including features of the basal leaves, umbellules, calyx teeth, and fruits.

Taxon	Note	Voucher	Locality	Basal leaves	Umbellules	Calyx teeth	Fruits	Figure
Sanicula serrata var. serrata	The type material of S. serrata var. serrata	*E.H. Wilson 156A* (K, NY-right part, US)	Hubei, Badong	The basal leaf segments with irregularly crenulate-serrate and subduplicately serrate margins throughout	The umbellules typically contain approximately six flowers, comprising five staminate and one fertile flower per umbellule	The calyx teeth are broadly ovate and shortly acuminate.	The fruits bear densely squamose spines, occasionally terminating in minute recurved projections, but are distinctly non-hooked	Fig. 1
	The type locality of S. serrata var. serrata (*LHM1111* population)	*Y.S. Zhang LHM1111* (NAS)	Hubei, Badong, Shennongxi	The basal leaves range from round-cordate to orbicular and are palmately divided into 3–5 segments. The central segment is obovate to cuneate-obovate, measuring 1.5–2 cm long and 2–3 cm broad, while the lateral segments are broadly obovate, typically divided to the middle or near the base, with finely serrate and irregularly subduplicately serrate margins	The umbellules contain 2–7 flowers, including 1–6 staminate flowers per umbellule	The calyx teeth are broadly ovate and shortly acuminate, ca. 0.7 mm long and 0.4 mm broad	During early fruiting, the fruits bear squamose spines; as they mature, the fruits become swollen and the spines transform into short, stiff projections, revealing a tuberculate-spiculate surface; mature fruits measure ca. 1.3 mm long and 1.7 mm broad.	Fig. 8
	The type material of S. tienmuensis var. tienmuensis	*West Lake Museum 67* (NAS, ZM)	Zhejiang, Hangzhou, Lin’an, West Tianmushan	The basal leaves that were cordate-rounded to orbicular, with unevenly short crenate-serrate margins bearing sharp teeth, occasionally entirely subduplicately serrate	Umbellules usually contain approximately six flowers, with five staminate and one fertile flower per umbellule	Calyx teeth are broadly ovate and shortly acuminate	Fruits are densely squamose, occasionally ending in minute recurved spines	Fig. 3A, B
	The type locality of Sanicula tienmuensis var. tienmuensis (*LHM1116* population)	*H.M. Li & L. Zhao 1116* (NAS)	Zhejiang, Hangzhou, Lin’an, west Tianmushan	The basal leaf blades range from round-cordate to orbicular, measuring 2–6 cm in length and 4–10 cm in breadth, palmately divided into 3–5 segments. The central segment is broadly obovate to cuneate-obovate (1.1–2.5 cm long, 2–5.5 cm broad), and the lateral segments are broadly obovate, typically divided to the middle or base, with irregularly and sharply serrate to subduplicately serrate margins	Umbellules generally contain 2–6 flowers, including 1–5 staminate and one fertile flowers per umbellule	Calyx teeth are broadly ovate, shortly acuminate, about 1 mm long and 0.7 mm broad	Immature fruits bear squamose spines; at maturity, fruits swell, developing short, stiff spines that reveal tuberculate-spiculate surfaces, measuring ca. 2.8 mm long and 3.7 mm broad	Fig. 12
	The type material of *S. elongata*	*P.C. Kuo 1424* (IBK, KUN, PE)	Shaanxi, Mei County, Laojunling	The basal leaves are subrounded to pentagonal, with irregularly and sharply serrate margins, occasionally entirely subduplicately serrate	Umbellules typically bear 4–6 flowers, comprising 3–5 staminate and one fertile flower per umbellule	Calyx teeth are narrowly ovate, approximately 0.3 mm long.	The fruits are ovoid, measuring about 3.0 mm long and 2.5 mm broad, and are densely covered with squamose spines, sometimes terminating in minute prickles.	Fig. 5A–C
	The population proximate to Mei County, the type locality of *S. elongata* (*LHM1207* population)	*H.M. Li & C.F. Song 1207* (NAS)	Shaanxi, Baoji City, Mount Zhongyan	The basal leaves observed in the field measured 2–5 cm long and 3–4 cm broad, ranging from round-cordate to pentagonal in outline, palmately divided into 3–5 segments, with irregularly and sharply serrate to occasionally subduplicately serrate margins	Umbellules contain 6–7 flowers, including 5–6 staminate and one fertile flower per umbellule	Calyx teeth are broadly ovate, shortly acuminate, approximately 0.5 mm long and 0.35 mm broad	Immature fruits bear squamose spines; at maturity, fruits swell, developing short, stiff spines that reveal tuberculate-spiculate surfaces, measuring approximately 1.6–2.2 mm long and 1.8–2.3 mm broad	Fig. 14
Sanicula serrata var. serrata	The type material of S. tienmuensis var. pauciflora	*Y.L. Cao 115* (CDBI)	Sichuan, Luding County, Desui Town, Mount Tianchi	The basal leaves are round-cordate to orbicular, palmately 3–5-parted, with margins irregularly and sharply serrate, occasionally subduplicately serrate throughout	Umbellules contain 2–5 flowers, including 1–4 staminate flowers and 1 fertile flower per umbellule	Calyx teeth are ovate to oblong-ovate, approximately 1.1 mm long and 0.8 mm broad	Fruits are ovoid, about 3.2 mm long and 3.1 mm broad, densely covered with squamose spines, and occasionally bear a small apical prickle.	Fig. 6
	The type locality of S. tienmuensis var. pauciflora (*LHM1167* population)	*H.M. Li & Y.S. Zhang 1167* (NAS)	Sichuan, Luding, Desui Town, Mount Tianchi	The basal leaves observed in the field measured 3–4 cm long and 5–8 cm broad, ranging from round-cordate to pentagonal in outline, palmately divided into 3–5 segments, with irregularly and sharply serrate to occasionally subduplicately serrate margins	Umbellules contain 1–7 flowers, including 0–6 staminate and one fertile flower per umbellule	Calyx teeth are broadly ovate, shortly acuminate, approximately 1 mm long and 0.55 mm broad	Immature fruits bear squamose spines; at maturity, fruits swell, developing short, stiff spines that reveal tuberculate-spiculate surfaces, measuring approximately 2.6–3.2 mm long and 2.8–3.4 mm broad	Fig. 17
	The type material of *S. langaoensis*	*B.N. Song, T. Ren & X.J. He SBN2023041201* (SZ)	Shaanxi, Lan’gao County	the basal leaves are round-cordate to orbicular, 0.8–3.2 cm long and 1.8–5.6 cm broad, palmately 3–5-parted, with margins irregularly and sharply serrate, occasionally subduplicately serrate throughout	Umbellules contain 5–6 flowers, including 4–5 staminate flowers and 1 sessile fertile flower per umbellule	Calyx teeth are ovate to oblong-ovate	Fruits are oblong-ovoid to ovoid, densely covered with squamose spines and occasionally have a small terminal prickle.	Fig. 7
*Sanicula potaninii*	The type material of *S. pataninii*	*G.N. Potanin s.n.* (LE)	Sichuan, Kangding	The basal leaves with distinctly crenate-serrate margins bearing attenuate-mucronate teeth	The umbellules contain 7–11 flowers, including 5–8 staminate flowers and 2–3 sessile fertile flowers per umbellule	The calyx teeth are broadly ovate and shortly acuminate, ca. 0.36 mm long and 0.25 mm broad	The fruits are ovoid and densely covered with uncinate spines	Fig. 2
	The type locality of *S. potaninii* and S. serrata var. uncinata (*LHM1163* population)	*H.M. Li & Y.S. Zhang 1163* (NAS)	Sichuan, Kangding County, Mount Paoma	The basal leaves with pentagonal-cordate to orbicular blades (ca. 5 cm long × 8 cm broad), palmately divided into three segments. The central segment is cuneate-obovate to cuneate (ca. 5 cm long × 2.5 cm broad), while the lateral segments are broadly cuneate-obovate and deeply bipartite, with crenate-serrate and attenuate margins	Umbellules contain 6–9 flowers, including 4–7 staminate and 1–3 fertile flowers per umbellule	The calyx teeth are broadly ovate with shortly acuminate to triangular- subulate, ca. 0.7–1.0 mm long, 0.38–0.42 mm broad	Fruits are prickly, measuring approximately 1.6 mm long and 2.5 mm broad, and are covered with purple uncinate spines	Fig. 9
	The type material of S. serrata var. uncinata	*Cunningham 37* (E)	Sichuan, Kangding	The basal leaves that are round-cordate to broadly pentagonal (0.9–3 cm long × 1.5–5.5 cm wide), palmately divided into 3–5 segments with crenate-serrate margins bearing attenuate-mucronate teeth	Umbellules typically contain 7–11 flowers, including 5–8 staminate and 2–3 fertile per umbellule	—	Fruits are ovoid and densely covered with uncinate spines.	Fig. 4

The basal leaf blades of the observed plants range from round-cordate to orbicular and are palmately divided into 3–5 segments. The central segment is obovate to cuneate-obovate, measuring 1.5–2 cm long and 2–3 cm broad, while the lateral segments are broadly obovate, typically divided to the middle or near the base, with finely serrate and irregularly subduplicately serrate margins (Fig. 8C). The umbellules comprise 2–7 flowers, including 1–6 staminate flowers. The calyx teeth are broadly ovate and shortly acuminate, approximately 0.7 mm long and 0.4 mm broad (Fig. 8I). During early fruiting, the fruits bear squamose spines; as they mature, the fruits become swollen and the spines transform into short, stiff projections, revealing a tuberculate-spiculate surface (Fig. 8H, I). Mature fruits measure approximately 1.3 mm long and 1.7 mm broad.

Analysis of the type material of *S. potaninii* (Fig. 2; Table 2) reveals basal leaves with distinctly crenate-serrate margins bearing attenuate-mucronate teeth. The umbellules contain 7–11 flowers, including 5–8 pedicellate staminate flowers arranged peripherally and 2–3 sessile fertile flowers clustered centrally. The fruits are ovoid and densely covered with uncinate spines (Fig. 2C, D). These diagnostic features were confirmed through field investigations at Mount Paoma, Kangding County, Sichuan – the type locality of *S. potaninii*.

**Figure 9.**
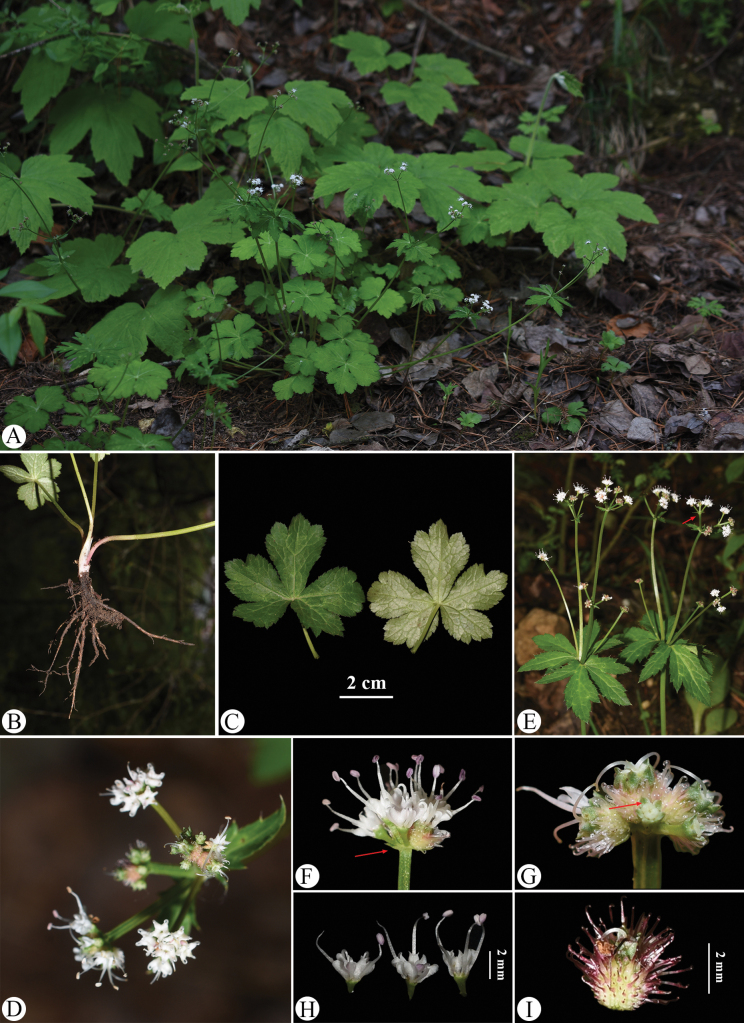
*Sanicula potaninii* in the wild (China, Sichuan, Kangding County, Mount Paoma, the type locality of *S. potaninii* and S. serrata var. uncinata). A. Habitat and habit; B. Rhizome; C. Leaf (left: adaxial surface; right: abaxial surface); D. Portion of the inflorescence (top view); E. Portion of the inflorescence (side view); F. Umbellule (side view), early fruiting, arrow indicating the involucellate bracteoles; G. Umbellule (side view), middle to late fruiting, arrow indicating the calyx teeth; H. Staminate flower (side view); I. Mericarps. Photographed by Hui-Min Li.

The observed plants possess basal leaves with pentagonal-cordate to orbicular blades (ca. 5 cm long × 8 cm broad), palmately divided into three segments. The central segment is cuneate-obovate to cuneate (ca. 5 cm long × 2.5 cm broad), while the lateral segments are broadly cuneate-obovate and deeply bipartite, with crenate-serrate and attenuate margins (Fig. 9C). Umbellules contain 6–9 flowers, including 4–7 staminate and 1–3 fertile flowers (Fig. 9F, G) per umbellule. The calyx teeth are triangular-subulate, ca. 1.0 mm long. Fruits are prickly, measuring approximately 1.6 mm long and 2.5 mm broad, and are covered with purple uncinate spines (Fig. 9I).

**Figure 10.**
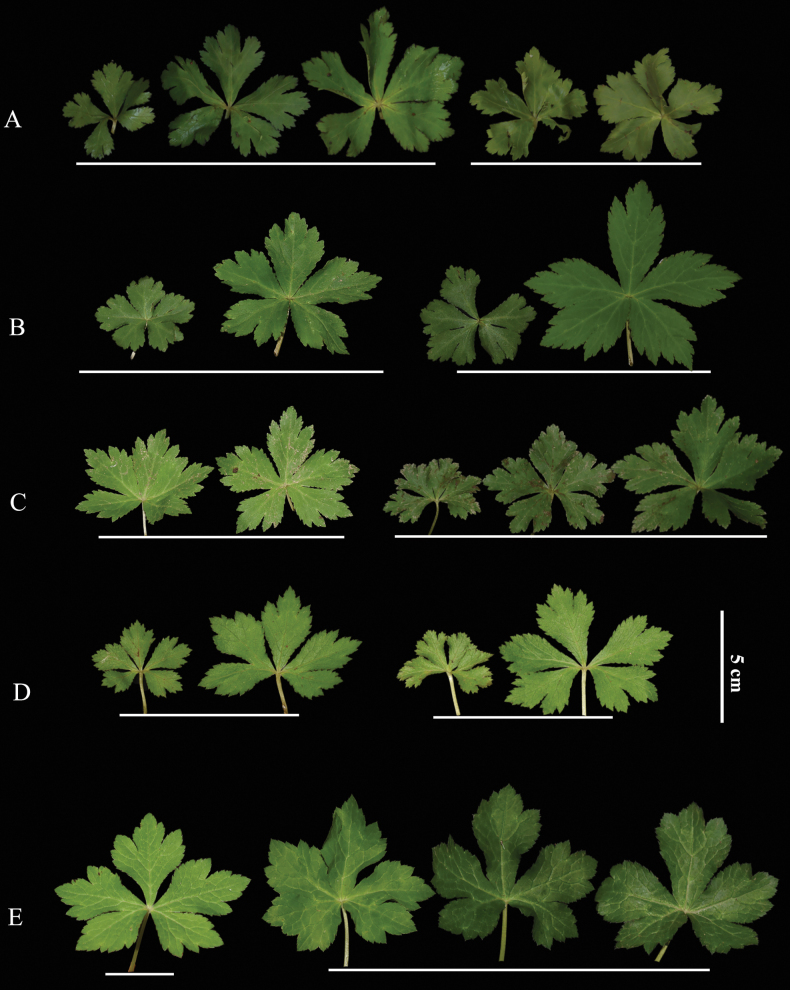
Basal leaves of Sanicula serrata var. serrata (A–D) and *S. potaninii* (E), showing variation in size and margin characteristics within and among populations. Leaves above the line come from the same plant individual. A. Hubei, Badong County, Shennongxi, *H.M. Li, Y.S. Zhang & Y. Xu 1110* (NAS); B. Zhejiang, Hangzhou, Lin’an, West Tianmushan, *H.M. Li & L. Zhao 1116* (NAS); C. Shaanxi, Baoji City, Mount Zhongyan, *H.M. Li & C.F. Song 1207* (NAS); D. Sichuan, Luding, Desui Town, Mount Tianchi, *H.M. Li & Y.S. Zhang 1167* (NAS); E. Sichuan, Kangding, Mount Paoma, *H.M. Li & C.F. Song 1200* (NAS). All at the same scale.

Field observations of four populations of S. serrata var. serrata indicated that the degree of leaf margin serration – ranging from blunt to sharp – varies both within and among populations, confirming its unreliability in distinguishing S. serrata var. serrata from *S. potaninii* (Fig. 10). For example, basal leaves of the Badong population (*LHM1111*) exhibit irregularly crenate to sharply serrate margins, occasionally subduplicate serrate throughout (Fig. 10A).

Specimen examinations and field observations (Fig. 9) further reveal that *S. potaninii* typically bears two to three fruits with densely uncinate spines, in clear contrast to S. serrata var. serrata, which usually produces a single fruit bearing squamose spines – occasionally tuberculate-spiculate or apically prickly. Consequently, fruit characteristics provide reliable diagnostic features for distinguishing these taxa. This evidence renders Pimenov’s (2017) treatment untenable; we therefore recognize *S. potaninii* as a distinct species. Sheh and Philippe (2005) reduced it to synonymy under *S. astrantiifolia* without justification; however, both herbarium specimens and living plants exhibit substantial differences in basal leaf morphology between *S. astrantiifolia* (Li et al. 2025) and *S. potaninii*.

**Figure 11.**
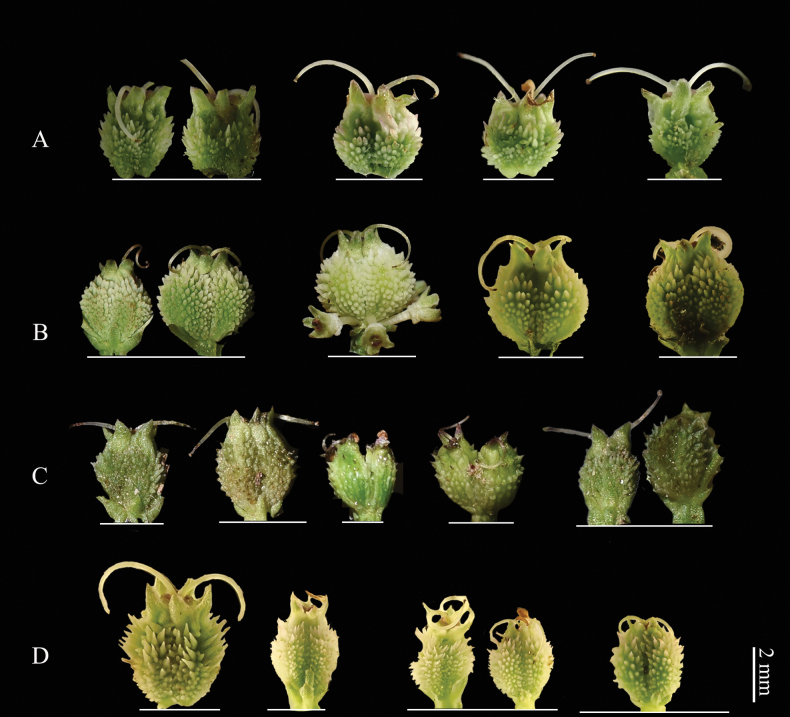
Fruits of Sanicula serrata var. serrata from four populations, showing variation in fruit characteristics. Each line represents an individual plant. A. Hubei, Badong County, Shennongxi, *H.M. Li, Y.S. Zhang & Y. Xu 1110* (NAS); B. Zhejiang, Hangzhou, Lin’an, West Tianmushan, *H.M. Li & L. Zhao 1116* (NAS); C. Shaanxi, Baoji City, Mount Zhongyan, *H.M. Li & C.F. Song 1207* (NAS); D. Sichuan, Luding, Desui Town, Mount Tianchi, *H.M. Li & Y.S. Zhang 1167* (NAS).

Notably, field observations of S. serrata var. serrata across Hubei Province and adjacent regions document individuals with immature fruits bearing tuberculate-squamose spines (Fig. 11A). As the fruits ripen, these spines elongate into squamose forms; at maturity, fruit enlargement is accompanied by spine shortening and stiffening, forming tuberculate-spiculate surfaces basally and hard, prickly surfaces apically – without ever developing hooked spines.

**Figure 12.**
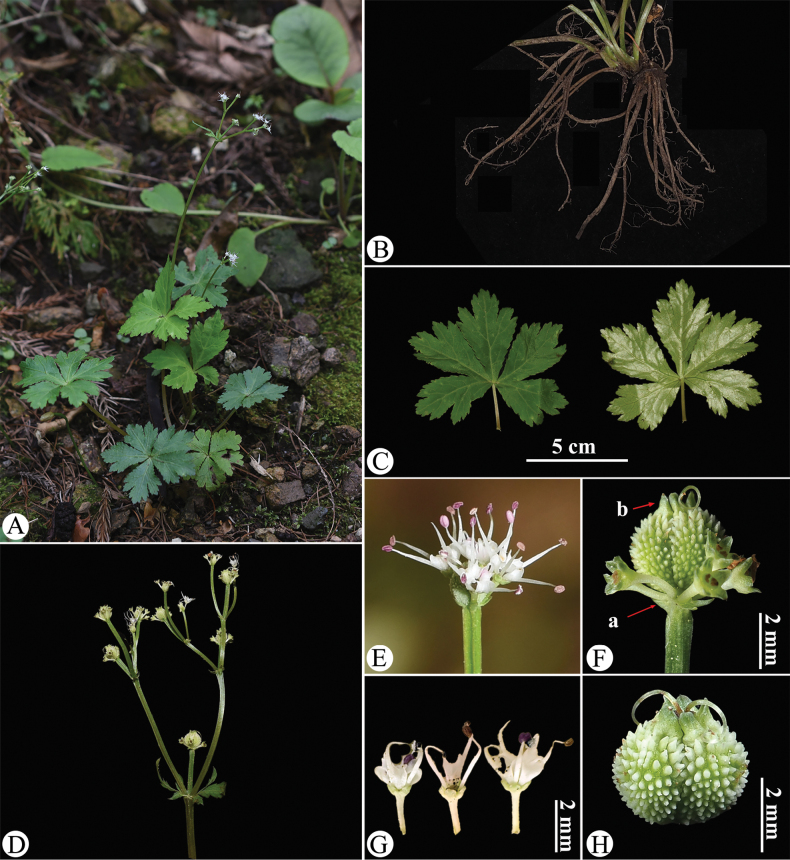
Sanicula serrata var. serrata in the wild (China, Zhejiang, Hangzhou, Lin’an, West Tianmushan, the type locality of S. tienmuensis var. tienmuensis). A. Habitat and habit; B. Rhizome; C. Leaf (left: adaxial surface; right: abaxial surface); **D** Portion of the inflorescence (side view); E. Umbellule (side view), early fruiting; F. Umbellule (side view), late fruiting, arrow (a) indicating the involucellate bracteoles; arrow (b) indicating the calyx teeth; G. Staminate flower (side view); H. Mericarps. Photographed by Hui-Min Li.

Our examination of the type material of S. tienmuensis var. tienmuensis (Fig. 3; Table 2) reveals that the basal leaves are cordate-rounded to orbicular with unevenly crenate-serrate to subduplicately serrate margins, umbellules usually bear ca. six flowers (typically five staminate and one fertile), broadly ovate and shortly acuminate calyx teeth, and densely squamose fruits sometimes terminating in minute recurved spines. These diagnostic characters were fully consistent with our field observations of living plants at the type locality in West Tianmushan, Zhejiang (Fig. 12; Table 2). Field observations further revealed that mature fruits become swollen, with short, stiff spines that expose a distinctly tuberculate-spiculate surface.

**Figure 13.**
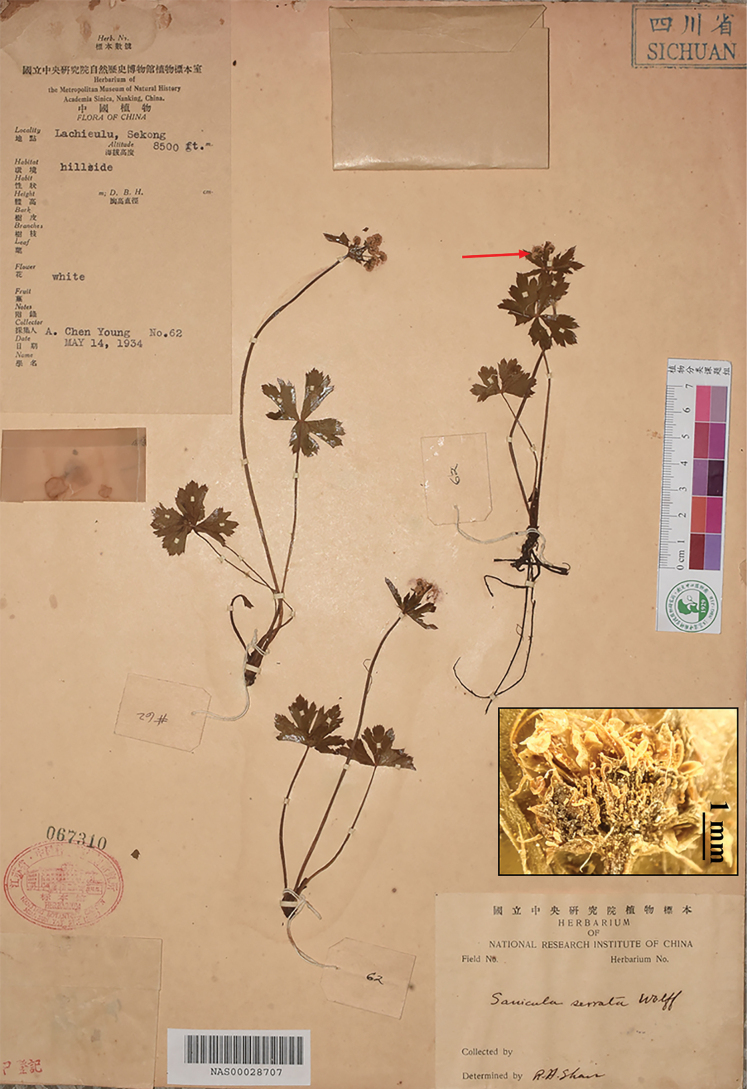
Specimen of *Sanicula potaninii*. Sichuan, Kangding, *A.C. Young 62* (NAS). Misidentified by Shan (1936) as *S. serrata.* Insert arrow indicates fruits with densely uncinate spines.

Our examination reveals clear morphological distinctions from *S. tuberculata* and *S. orthacantha* (Li et al. 2022). However, Shan and Constance (1951) previously noted differences between *S. tienmuensis* and S. serrata var. serrata based on fruit characters. It is noteworthy that comprehensive understanding of S. serrata var. serrata has been hindered by insufficient specimen examination and lack of field observations. For example, Shan (1936) identified specimen *A.C. Young 62* (NAS00028707; Fig. 13) from Kangding, Sichuan, as S. serrata var. serrata, whereas our analysis confirmed this specimen represents *S. potaninii*. Therefore, morphologically, S. tienmuensis var. tienmuensis is indistinguishable from S. serrata var. serrata, indicating they should be treated as conspecific.

Examination of the type material of S. serrata var. uncinata (Fig. 4; Table 2) reveals that the basal leaves are round-cordate to broadly pentagonal (0.9–3 cm long × 1.5–5.5 cm wide), palmately divided into 3–5 segments with crenate-serrate margins bearing attenuate-mucronate teeth. Umbellules typically contain 7–11 flowers (5–8 staminate and 2–3 fertile per umbellule). Fruits are ovoid and densely covered with uncinate spines. These morphological features are corroborated by field observations of living plants (Fig. 9; Table 2) from Mount Paoma, Kangding County, Sichuan – the type locality.

Comparative analysis indicates no substantial morphological differences between S. serrata var. uncinata and *S. potaninii*. Both taxa consistently exhibit umbellules bearing 2–3 fertile flowers and fruits densely covered with uncinate spines (Fig. 9G, I). Given the congruence in vegetative and reproductive traits, these two entities do not warrant taxonomic distinction.

**Figure 14.**
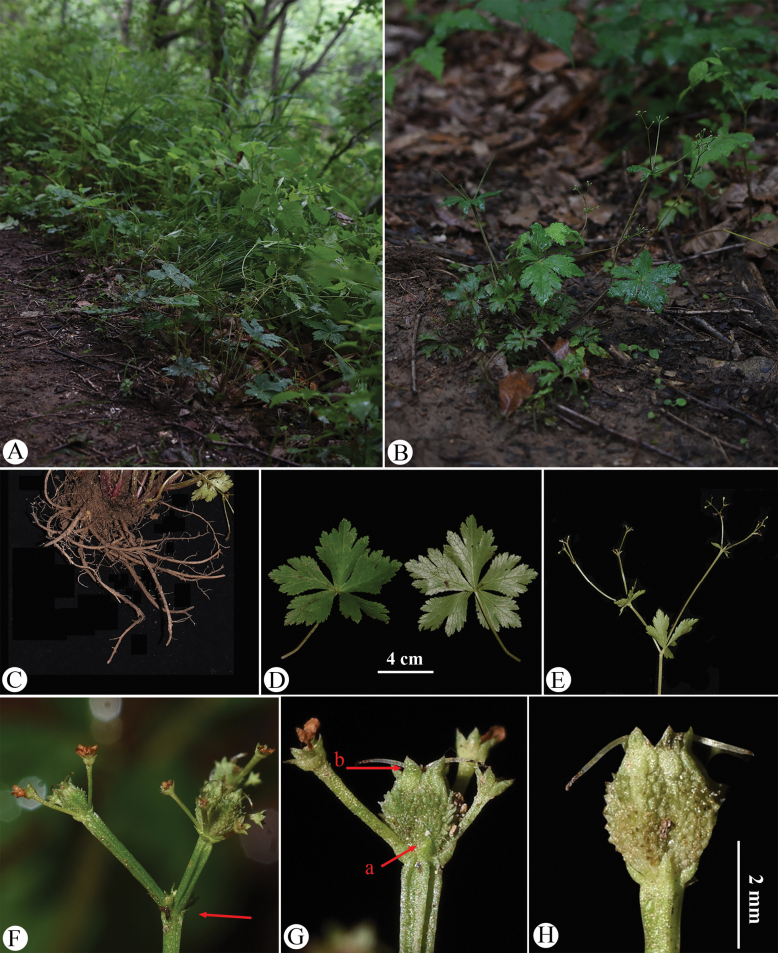
Sanicula serrata var. serrata in the wild (Shaanxi, Baoji City, Mount Zhongyan, near the type locality of *S. elongata*). A. Habitat; B. Habit; C. Rhizome; D. Leaf (left: adaxial surface; right: abaxial surface); E. Portion of the inflorescence (side view); F. Portion of the inflorescence (side view), arrow indicating the involucral bract; G. Umbellule (side view), late fruiting, arrow (a) indicating the involucellate bracteoles; arrow (b) indicating the calyx teeth; H. Mericarps. Photographed by Hui-Min Li.

Our examination of the type material of *S. elongata* (Fig. 5; Table 2) reveals that the basal leaves are subrounded to pentagonal, with irregularly and sharply serrate margins, occasionally entirely subduplicately serrate. Umbellules typically bear 4–6 flowers, comprising 3–5 staminate flowers and one fertile flower per umbellule. Calyx teeth are narrowly ovate. The fruits are ovoid and densely covered with squamose spines, sometimes terminating in minute prickles. These morphological features are corroborated by field observations of living plants (Fig. 14; Table 2) collected from Mount Zhongyan, Weibin, Baoji, northwestern Shaanxi – proximate to Mei County, the type locality of *S. elongata*. The basal leaves observed in the field range from round-cordate to pentagonal in outline, palmately divided into 3–5 segments, with irregularly and sharply serrate to occasionally subduplicately serrate margins (Fig. 14D). Umbellules contain 6–7 flowers, including 5–6 staminate flowers and one fertile flower per umbellule. Calyx teeth are broadly ovate, shortly acuminate (Fig. 14G, H). Immature fruits bear squamose spines; at maturity, fruits swell, developing short, stiff spines that reveal tuberculate-spiculate surfaces (Fig. 14H).

**Figure 15.**
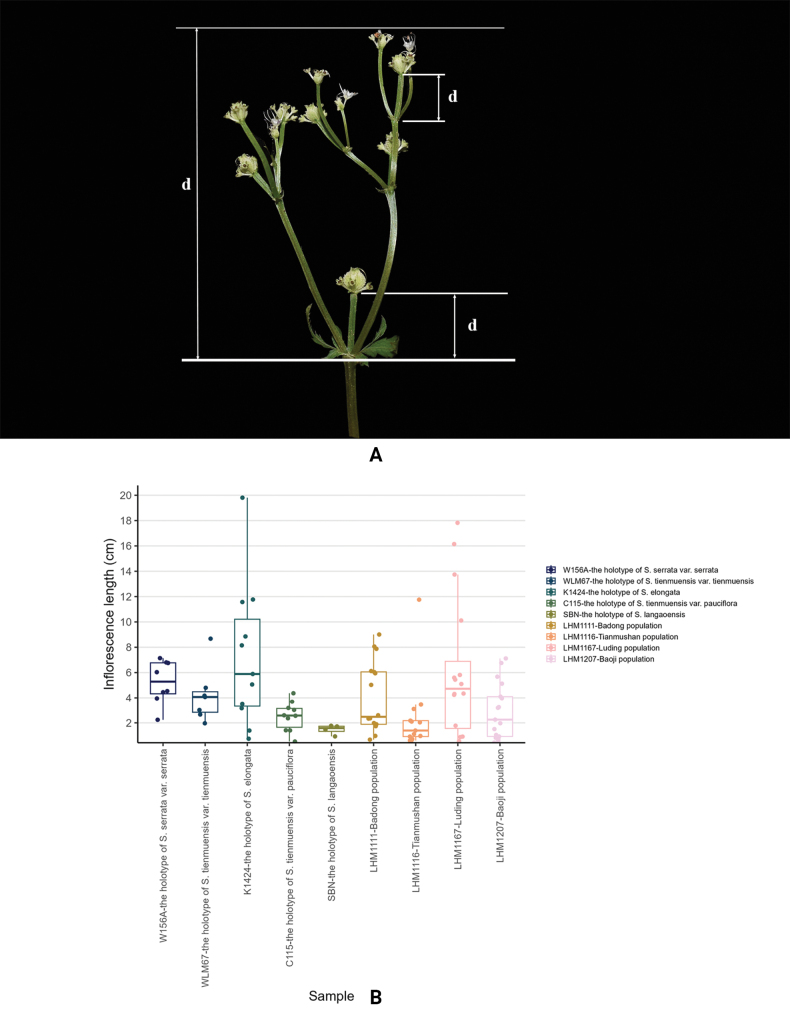
Variation in inflorescence length across nine populations of Sanicula serrata var. serrata. A. Measurement of inflorescence length, from the first visible umbel to the terminal umbel on each individual; B. Boxplots showing variation across populations. Filled circles represent individual measurements; boxes indicate interquartile ranges (IQR, Q1–Q3); horizontal lines denote medians; whiskers extend to ± 1.5× IQR; outliers beyond whiskers are shown individually. The presence of continuous intraspecific variation is evidenced by overlapping value ranges and clinal transitions among populations.

A numerical analysis of nine field-sampled populations of *Sanicula* in this study revealed considerable variation in inflorescence length both within and among populations (Suppl. material 1; Fig. 15). Furthermore, fruit spine morphology was found to be highly variable across and within these populations (Fig. 11). Importantly, detailed examination of herbarium specimens (including type material), combined with field observations, indicates that variation in features related to reproductive success, particularly fruit set, should not be considered a diagnostic character within *Sanicula*. Consequently, no significant morphological distinctions exist between *S. elongata* and S. serrata var. serrata, especially with respect to inflorescence length and fruit characteristics (Figs 5, 11).

**Figure 16.**
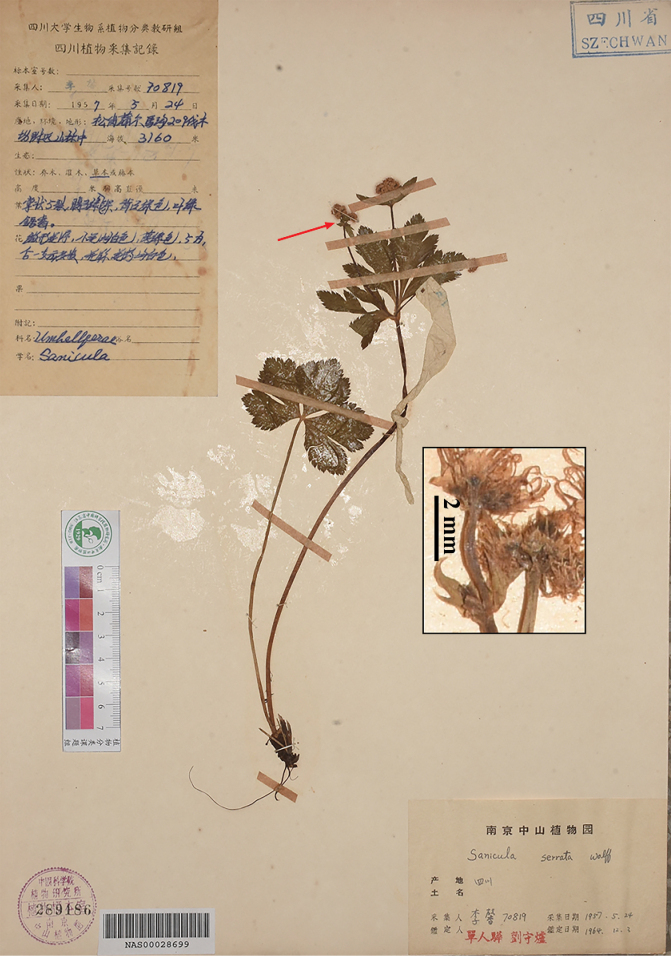
Specimen of *Sanicula potaninii*. Sichuan, Markang City, Pu’erma Village, *X. Li 70819* (NAS). Pu (1993) misidentified as *S. serrata.* Insert arrow indicates fruits with densely purple uncinate spines.

As noted above, Wu (1993), likely influenced by Shan (1936), recognized S. serrata var. serrata in the Hengduan Mountains. Among the three specimens he cited, *X. Li 70819* (KUN0465723, PE00754987, NAS00028699, NAS00578947, SZ00129115; Fig. 16; here represented by NAS00028699) actually refers to *S. potaninii*. Therefore, his (1979) recognition of *S. elongata* and comparison with S. serrata var. serrata, particularly his assertion that the latter possesses hooked spines, was likely influenced by earlier taxonomic misinterpretations.

**Figure 17.**
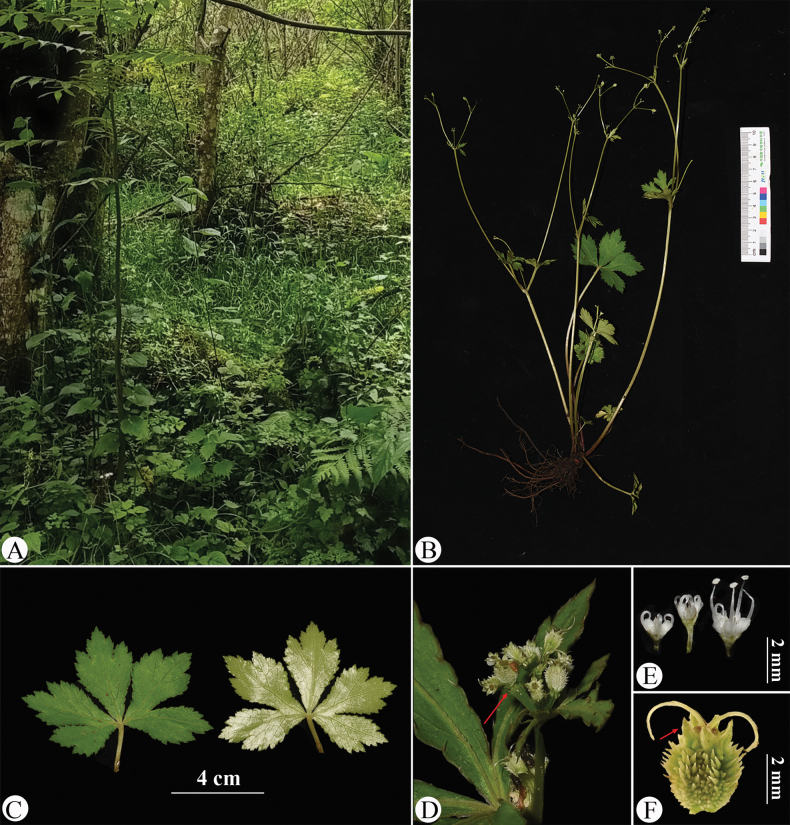
Sanicula serrata var. serrata in the wild (Sichuan, Luding, Desui Town, Mount Tianchi, the type locality of S. serrata var. pauciflora). A. Habitat; B. Habit; C. Leaf (left: adaxial surface; right: abaxial surface); D. Portion of the inflorescence (side view), arrow indicating the involucellate bracteoles; E. Staminate flower (side view); F. Mericarps, arrow indicating the calyx teeth. Photographed by Hui-Min Li.

Our examination of the type material of S. tienmuensis var. pauciflora (Fig. 6; Table 2) reveals that the basal leaves are round-cordate to orbicular, palmately 3–5-parted, with margins irregularly and sharply serrate, occasionally subduplicately serrate throughout. Umbellules contain 2–5 flowers (1–4 staminate and one fertile). Calyx teeth are ovate to oblong-ovate, and fruits are ovoid, densely covered with squamose spines, occasionally with a small, apical prickle. These features are corroborated by our field observations of living plants from Mount Tianchi, Luding County, Sichuan – the type locality (Fig. 17; Table 2). The basal leaves observed in the field range from round-cordate to pentagonal in outline, palmately divided into 3–5 segments with similarly serrate margins (Fig. 17C). Umbellules contain 1–7 flowers (0–6 staminate and one fertile flower). Calyx teeth are broadly ovate and shortly acuminate. Immature fruits bear squamose spines; at maturity, fruits swell, developing short, stiff spines that reveal tuberculate-spiculate surfaces (Fig. 17F).

It is also noteworthy that in our field investigations across Anhui, Hubei, Sichuan, Shaanxi, Yunnan, and Zhejiang Provinces in recent years, we consistently observed a pattern in the relative species of *Sanicula* L. where each umbellule initially bears five to six staminate flowers. However, as the plants mature, the staminate flowers are shed, resulting in umbellules that retain only zero to three staminate flowers at anthesis. This phenomenon indicates that the reduced number of staminate flowers observed in S. tienmuensis var. pauciflora is not taxonomically significant but rather depends on the developmental stage. Therefore, our observations suggest no essential morphological distinction between S. tienmuensis var. pauciflora and S. serrata var. serrata, particularly regarding fruit morphology and the number of staminate flowers per umbellule.

The analysis of type material and images of living plants provided in the protologue of *S. langaoensis* (Fig. 7; Table 2) reveals that the basal leaves are round-cordate to orbicular, 0.8–3.2 cm long and 1.8–5.6 cm broad, palmately 3–5-parted, with margins irregularly and sharply serrate, occasionally subduplicately serrate throughout. Umbellules bear 5–6 flowers, including 4–5 staminate flowers and 1 sessile fertile flower per umbellule. Calyx teeth are ovate to oblong-ovate. Fruits are oblong-ovoid to ovoid, densely covered with squamose spines and occasionally have a small, terminal prickle.

Upon careful examination of the images presented in the protologue (Song et al. 2024), we found that the authors’ claim of 9–10 staminate flowers per umbellule is inconsistent with the actual floral morphology depicted. Moreover, the protologue lacks any comparative analysis with morphologically similar taxa occurring in the same geographical region. Based on these findings, we conclude that *S. langaoensis* is morphologically indistinguishable from S. serrata var. serrata and thus should not be considered a separate taxon.

In conclusion, S. serrata var. serrata can be distinguished from *S. potaninii* by its fruit characteristics: the former typically has a single fruit densely covered with squamose spines, occasionally exhibiting tuberculate-spiculate or apically prickly surfaces, whereas the latter usually bears two to three fruits per umbellule, each densely adorned with uncinate spines. Taking into account differences in geographic distribution, elevational range, and key morphological traits within the genus, it is appropriate to recognize these two taxa as distinct species.

Moreover, our comprehensive examination of herbarium specimens (including type material) and living plants, supported by numerical analyses of inflorescence length, staminate flower number, and fruit character, indicates that *S. elongata*, *S. langaoensis*, S. serrata var. serrata, S. tienmuensis var. tienmuensis, and S. tienmuensis var. pauciflora are morphologically indistinguishable. Similarly, no consistent morphological distinctions are observed between *S. potaninii* and S. serrata var. uncinata.

Accordingly, we synonymize *S. elongata*, *S. langaoensis*, S. tienmuensis var. tienmuensis, and S. tienmuensis var. pauciflora with S. serrata var. serrata, and place S. serrata var. uncinata in synonymy with *S. potaninii*.

### ﻿Taxonomic treatment

#### ﻿1. Sanicula serrata

Taxon classificationPlantaeApialesApiaceae

H. Wolff in Engler, Pflanzenr. 61: 56 (1913).

661E2FD7-700C-55E6-84F1-7B3CA5D5E1EF

Figs 1, 3, 5, 6, 7, 8, 12, 14, 17 锯叶变豆菜

= Sanicula tienmuensis R.H. Shan & Constance, Univ. Calif. Publ. Bot. 25: 23 (1951), syn. nov. Type: China. Zhejiang, Hangzhou, Lin’an, West Tianmushan, 30 April 1931, *West Lake Museum 67* (holotype: NAS00082802!; isotypes: ZMNH0057084!, ZMNH0057085-right part!).
= Sanicula elongata K.T. Fu, Fl. Reipubl. Popularis Sin. 55(1): 297 (1979), syn. nov. Type: CHINA. Shaanxi, Mei County, Laojunling, 17 June 1952, *P.C. Kuo 1424* (lectotype: IBK00159684! designated here; isolectotypes: IBK00159685!, IBK00159686!, KUN0465506!, PE01933863!).
= Sanicula tienmuensis var. pauciflora R.H. Shan & F.T. Pu, Acta Phytotax. Sin. 27(1): 66 (1989), syn. nov.
≡ Sanicula pauciflora (R.H. Shan & F.T. Pu) B.N. Song & X.J. He, Frontiers Pl. Sci. (Online journal) 15–1351023: 12 (2024).
Type: CHINA. Sichuan, Luding County, Desui Town, Mount Tianchi, under forests or by streams, alt. 2,200 m, 1 May 1984, *Y.L. Cao 115* (lectotype: CDBI0172308! designated here; isolectotype: CDBI0172309!).
= Sanicula langaoensis B.N. Song, T. Ren & X.J. He, Frontiers Pl. Sci. (Online journal) 15–1351023: 13 (2024), syn. nov. Type: China. Shaanxi, Lan’gao County, in stream banks inmixed forests, 32°13'47.88"N, 108°53'45.18"E, alt. 1,496 m, 12 April 2023, *B.N. Song, T. Ren & X.J. He SBN2023041201* (holotype: SZ!).

##### Type.

China. • Hubei, Badong (= Patung), April 1900, *E.H. Wilson 156a* (lectotype: K001325368!, designated by Pimenov (2017); isolectotype: NY00406258-right lower part!, US00126980!). Fig. 1.

**Description.** Perennial. Rhizome short and stout, roots fascicled, fleshy, with fibrous. Stem 1–4, slender and erect, several branched above the middle, 7–47 cm tall. Basal leaves 2–8, long petiolate; petioles 1.5–23 cm long; blade glabrous adaxially and abaxially, 1–7 cm long, 1.5–16.5 cm wide, rounded-cordate to cordate-pentagonal, tripartite to the base or trifoliate, the median segment cuneate-obovate to rhombic-cuneate, distinctly shallowly trilobed, the lateral segments oblique, usually bilobed below the middle or nearly to the base, the margins irregularly crenulate-serrate with sharply serrate teeth. Cauline leaves few, conspicuous, ternate, upper stem leaves subsessile or sessile, tripartite, resembling involucrate bract, (0.5–)1.5–3 cm long, (0.3–)1.3–2.5(–4.5) cm wide. Inflorescence 1–3-branched; involucrate bract usually 2, trisect, 1–6 mm long; rays of the umbels 1–6, 2–13 mm long; involucellate bracteoles 4–6, oblong ovate, 0.2–0.8 mm long. Umbellules 1–8-flowered, staminate flowers 0–7 per umbellule, pedicels 1.9–3.2 mm long, petals white, fertile flowers 1 per umbellule, sessile; calyx teeth oblong ovate, 0.5–0.9 mm long; styles 1.9–3.0 mm long, 2–5 times longer than the calyx teeth. Mericarps oblong-ovoid to ovoid, 1.8–2.6 mm long, 1.3–3 mm broad, tuberculate spiculate and occasionally with prickly spines on the top during later ripening. Vittae obsure.

##### Distribution.

**Figure 18.**
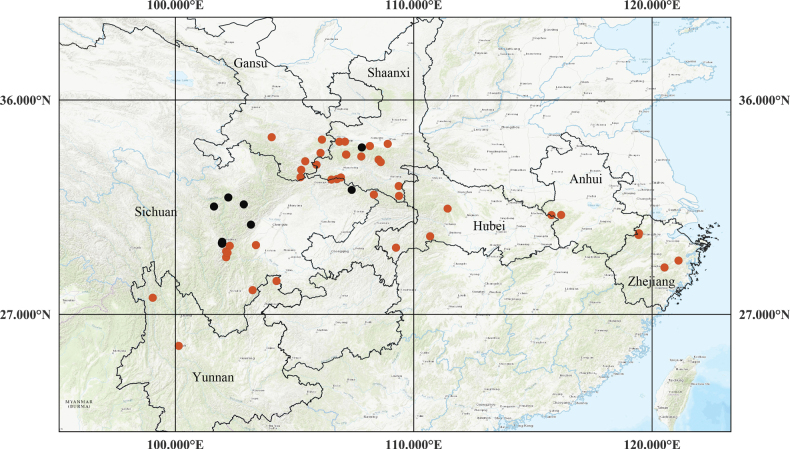
Distribution of Sanicula serrata var. serrata (red circles) and *S. potaninii* (black circles).

*Sanicula serrata* is widely distributed in China (Anhui, Gansu, Hubei, Shaanxi, Sichuan, Yunnan and Zhejiang) (Fig. 18).

##### Habitat.

This species grows on mountain slopes under forest or along ravine streams near rock or along the road under forest at elevations of 500–3,200 m above sea level.

##### Phenology.

Flowering from April to May, fruiting from April to June.

##### Etymology.

The epithet serrata is derived from Latin and refers to the serrated margins of the leaves.

##### Additional specimens examined.

**Anhui Province**, • Huoshan [Hoshan] County, Cangping Village, 31°10'29.9"N, 116°11'6.3"E, alt. 796 m, 10 April 2021, *H.M. Li, Y.S. Zhang, Y. Xu 1110* (NAS); • Huoshan County, Cangpingdagou, alt. 950 m, 27 March 1982, *M.B. Deng 81269* (NAS); • Huoshan County, Cangpingdagou, alt. 1,000 m, 9 June 1980, *M.B. Deng, K. Yao 80870* (NAS); • Jinzhai [Chin Chai] County, Baima Zhai Forest Farm, alt. 650 m, 19 April 1993, *K.Y. Lang, et al. 930047* (PE); • Jinzhai County, Baima Zhai Forest Farm, alt. 900 m, 19 May 1984, *K. Yao 9027* (NAS); • Jinzhai County, Baima Zhai Forest Farm, alt. 1,000 m, 17 May 1984, *M.B. Deng 81743* (NAS); • Jinzhai County, Baima Zhai Forest Farm, alt. 1,100 m, 14 May 1984, *Anonymous 81722* (NAS); • Jinzhai County, Baima Zhai Forest Farm, alt. 1,300 m, 14 May 1984, *M.B. Deng 81721* (NAS); •Jinzhai County, Baimazhai, alt. 1,050 m, 21 April 1985, *X.S. Shen 218* (PE). **Gansu Province**, • Hui County, alt. 1,800 m, 30 June 1956, *P.C. Kuo 3167* (WUK); • Kang County, Changba Township, alt. 1,600 m, 23 April 1963, *Y.Q. He, C.L. Tang 31* (WUK); • Kang County, alt. 1,500 m, 3 May 1963, *Y.Q. He, C.L. Tang 249* (WUK); • Long’an City, Wudu District, Luotang Town, Yuezhao Township, alt. 2,400 m, 24 June 1959, *Z.Y. Zhang 5687* (LBG, WUK); • Min County, alt. 3,200 m, 16 June 1951, *T.P. Wang 15279* (KUN, NAS, WUK); • Tianshui County, Dangchuan, alt. 2,200 m, 22 June 1964, *K.T. Fu 15559* (WUK); • Tianshui County, 1,200 m, 16 April 1959, *S.B. He 1048* (WUK); • Wen County, Bikou Town, alt. 700 m, 24 March 1972, *T.P. Wang 20442* (WUK). **Hubei Province**, • Badong County, Shennongxi, *Y.S. Zhang LHM1111* (NAS); • Nanzhang County, Xueping Town, 29°48'07.4"N, 109°15'25.1"E, alt. 742 m, 16 May 2025, *F. Tan LHM1701* (NAS); • Wufeng County, Xiaolong Village, 30°16'44.4"N, 110°41'20.4"E, alt. 1,657 m, 18 May 2025, *F. Tan LHM1700* (NAS); • Baoji City, Weibin District, Jifengshan, alt. 1,650 m, 28 May 1977, *Z.X. Hu, Y.H. Guo 313* (WUK); • Baoji City, Mount Zhongyan, 34°15'10.9"N, 107°06'56"E, alt. 1,778 m, 3 June 2022, *H.M. Li, C.F. Song 1207* (NAS); • Chang’an County, 1956, *Huanghe Exped. 360* (KUN); • Feng County, Miaowangshan, alt. 2,400 m, 12 June 1977, *K.T. Fu 17337* (WUK); • Foping County, Donghe Village, alt. 2,100 m, 11 May 2008, *S.F. Li, B. Li, Y. Zhang, et al. 10192* (XBGH); • Foping County, Gaojiaba, alt. 1,800 m, 20 July 1952, *K.T. Fu 5115* (KUN, IBK, WUK); • Liuba County, Miaotaizi Forest Farm, 22 April 1982, *Z.Y. Zhang 18454* (WUK); • Liuba County, Sangyuanba Township, 33°42.720'N, 107°9.635'E, alt. 1,072–1,470 m, 28 May 2010, *S.F. Li, B. Li, Y. Zhang, et al. 13530* (XBGH); • Liuba County, 33°42'25.8"N, 107°10'12.4"E, alt. 1,366 m, 4 June 2022, *H.M. Li, C.F. Song 1219* (NAS); • Lueyang County, Miaogou, 33°17'11.4"N, 105°55'17.8"E, alt. 1,295 m, 5 June 2022, *H.M. Li, C.F. Song 1224* (NAS); • Lueyang County, Zengjiagou, 33°16'59.6"N, 105°55'19.4"E, alt. 1,380 m, 5 June 2022, *H.M. Li, C.F. Song 1223* (NAS); • Lueyang County, Zhangjiaba, alt. 1,300 m, 4 May 1957, *The Fifth Forest Survey Team of the Ministry of Forestry 9* (WUK); • Nanzhen County, Longdonggou, 32°44'56.8"N, 106°56'45.0"E, alt. 869 m, 6 June 2022, *H.M. Li, C.F. Song 1227* (NAS); • Nanzhen County, On the roadside from Xihe to Xiaoba, *X.X. Hou 226* (NAS); • Nanzhen County, Xiaoba, alt. 1,973 m, 20 April 1973, *X.X. Hou 475* (WUK); • Ningshan County, Guankou, alt. 1,800 m, 3 June 1959, *J.Q. Xing 4388* (NAS, WUK); • Ningshan County, Guankou Town, alt. 1,690 m, 30 May 1959, *J.Q. Xing 4049* (NAS, SZ, WUK); • Ningshan County, Guankou Town, alt. 1,925 m, 4 June 1959, *J.Q. Xing 5661* (SZ, WUK); • Ningshan County, Xunyangba, alt. 1,439 m, 13 May 2013, *X.H. Tian, L.H. Wu, TianXH823* (KUN); • Ningshan County, Xunyangba, 24 April 1993, *X.H. Tian, L. Zhang T934021* (PE); • Ningshan County, Zhifanggou, 33°29'48.9"N, 108°31'32.3"E, alt. 1,679 m, 9 June 2022, *H.M. Li, C.F. Song 1239* (NAS); • Pingli County, Baxian Town, 32°1.720'N, 108°19.690'E, alt. 1,900 m, 8 April 2004, *Y.S. Chen, Z.H. Wu, B. Li et al. 275* (WUK); • Zhen’an County, Lizha Forest Farm, alt. 1,830 m, 30 May 1973, *X.X. Hou, Y.H. Guo 650* (WUK); • Zhen’an County, Muwang Forest Farm, 33°23.712'N, 108°37.017'E, alt. 2,037 m, 9 June 2008, *S.F. Li, B. Li, Y. Zhang, et al. 10475* (XBGH); • Zhen’an County, Muwang Forest Farm, 33°23'43.4"N, 108°37'01.5"E, alt. 2,015 m, 8 June 2022, *H.M. Li, C.F. Song 1232* (NAS); • Zhenping County, Falong Village, 31°58'35.3"N, 109°23'02.4"E, alt. 1,637m, 7 June 2022, *H.M. Li, C.F. Song 1231* (NAS); • Zhenping County, Shangzhu Township, alt. 1,710 m, 3 May 1989, *G.Y. Xu 4641* (WUK); • Zhenping County, 1970, *Zhenping Pharma. Exped. 111* (WUK); • Zhouzhi County, Xinkou, alt. 2,000 m, 13 June 1952, *P.C. Kuo 1313* (IBK, KUN, WUK); • Zhouzhi County, alt. 1,220 m, 25 April 1977, *Z.X. Hu, Y.H. Guo 89* (WUK); • Pingli County, alt. 1,820 m, 27 May 1959, *P.Y. Li 2566* (KUN, WUK). **Sichuan Province**, • Hongya [Hung ya] County, Hongya Forest Farm, Mahuanggou, alt. 1,950 m, 27 June 1994, *W.K. Bao, et al. 2485* (CDBI); • Luding County, Desui Town, Tianchishan, 29°35'20.4"N, 102°11'16.5"E, alt. 2,355 m, 18 May 2021, *H.M. Li, Y.S. Zhang 1167* (NAS); • Luding County, Moxi Town, alt. 2,350 m, 8 April 1981, *Sichuan Plant Exped. 25023* (CDBI); • Luding County, Moxi Town, alt. 2,900 m, 8 June 1980, *Q.Q. Wang, Z.A. Liu 22183* (CDBI); • Nanjiang County, Guangming Township, Guangwushan, alt. 1,600 m, 5 June 1956, *Sichuan-Da Econ. Plant Exped. 2675* (KUN); • Nanjiang County, alt. 1,400–1,500 m, 5 June 1959, *B.L. Chen 2558* (KUN); • Shimian County, Caoke Township, alt. 2,300 m, 1978, *Shimian Exped. 78–1149* (SM); • Shimian County, alt. 1,850 m, 11 April 1955, *C.J. Xie 39763* (PE); • Shimian County, alt. 1,850 m, 11 April 1955, *C.J. Xie 39763* (PE, WUK); • Tianquan County, Erlangshan, alt. 1,550 m, 20 April 1956, *D.P. He 43222* (SZ); • Sichuan, Tianquan County, Erlangshan, alt. 1,700–2,050 m, 26 April 1986, *T. Naito et al. 150* (PE); • Tianquan County, Lianghe Township, alt. 1,600 m, 22 April 1956, *D.P. He 42968* (SZ); • Tianquan County, Xingou, alt. 1,700 m, 3 June 1959, *Sichuan-Ya Econ. Plant Exped. 551* (SM); • Tianquan County, Xingou Village, alt. 1,720 m, 1 April 1980, *Y.B. Yang 21603* (CDBI); • Tianquan County, alt. 1,300 m, 23 April 1956, *D.P. He 43013* (SZ); • Tianquan County, alt. 1,550 m, 21 April 1956, *D.P. He 42940* (SZ); • Tianquan County, alt. 1,800 m, 31 March 1953, *X.L. Jiang 33663* (PE, SZ); • Tianquan County, alt. 2,800 m, 6 May 1978, *Tianquan Exped. 78–132* (SM); • Tianquan County, 1936, *K.L. Chiu 2300* (NAS, PE); • Tianquan County, *K.L. Chiu 2398* (PE); • Wangcang County, Micangshan Nature Reserve Shiziba Conservation Station, 32°39'42.3"N, 106°32'48.2"E, alt. 1,651 m, 28 June 2011, *Bashan Exped. 4815* (PE); • Zhaojue County, Guli Town, alt. 1,280 m, 22 April 1979, *Zhaojue Exped. 96* (SM); • Sichuan, Zhaojue County, Guli Town, alt. 1,720 m, 24 April 1979, *Zhaojue Exped. 133* (SM). **Yunnan Province**, • Dali City, Fengyandong, June 1941, *H.C. Wang 804* (PE); • Gongshan County, 28°24'06.7"N, 104°14'37.3"E, alt. 2,049 m, 1 April 2023, *H.M. Li, J.W. Zhu, B.C. Wu 130* (NAS); • Weixi County, Baimaluo, alt. 2,500 m, 2 June 1940, *K.M. Feng 4366* (KUN, PE); • Weixi County, Yezhi Town, alt. 2,700 m, 8 May 1982, *Qinghai-Tibet Plateau Exped. 6330* (KUN, PE); • Weixi County, alt. 2,600 m, 29 April 1940, *K.M. Feng 3543* (KUN, PE); • Weixi County, alt. 3,500 m, June 1935, *C.W. Wang 63892* (PE). **Zhejiang Province**, • Anji County, Longwangshan, 30°24'29.44"N, 119°26'59.21"E, alt. 933 m, 16 April 2021, *H.M. Li, L. Zhao 1114* (NAS); • Anji County, Longwangshan, alt. 500 m, 14 March 1997, *L.P. Yu, M.B. Deng 97025* (PE); • Anji County, Longwangshan, alt. 600 m, 16 May 1995, *M.B. Deng 90144* (PE); • Anji County, Longwangshan, 3 May 1988, *Anonymous 1718* (ZM); • Anji County, Longwangshan, Qianrenqiao, alt. 1,100 m, 3 May 1987, *Xu 281* (ZM); • Anji County, Longwangshan, Xianrenqiao, alt. 550 m, 7 May 1997, *Y.M. Fang, M.B. Deng 975152* (PE); • Anji County, Xiaofeng Town, 1958, *H.Y. Ho 24193* (HZ, NAS, PE); • Hangzhou City, Lin’an District, West Tianmushan, 30°20'13"N, 119°26'2"E, alt. 777 m, 17 May 2021, *H.M. Li, L. Zhao 1116* (NAS); • Hangzhou City, Lin’an District, West Tianmushan, 30°20'13"N, 119°26'2"E, alt. 783 m, 20 April 2025, *X.D. Ma 360* (NAS); • Hangzhou City, Lin’an District, Tianmushan, alt. 580 m, 25 April 1957, *H.Y. Ho 21100* (HZ, NAS); • Hangzhou City, Lin’an District, West Tianmushan, alt. 800 m, 25 May 1957, *Yuan et al. 4114* (HHBG, NAS, WUK); • Hangzhou City, Lin’an District, West Tianmushan, 23 April 1936, *H. Migo s.n.* (NAS); • Hangzhou City, Lin’an District, *Anonymous 94* (HZ); • Pan’an County, Dapanshan, 28°58'17.91"N, 120°31'17.05"E, alt. 1,104 m, 18 April 2021, *H.M. Li, L. Zhao 1117* (NAS); • Tiantai County, Huadingshan, 21 April 1955, *T.Y. Cheo, J.S. Yue 978* (NAS).

##### Notes.

**1)** In the protologue of *Sanicula serrata*, Wolff (1913) indicated that the type collection, *E.H. Wilson 156a*, was deposited at B. This specimen was most likely destroyed during World War II. Five duplicates of this collection are currently preserved at E, HBG, K, NY, and US, respectively, and are all in good condition. However, upon detailed examination of both herbarium material and living plants, we observed that the characteristics of the cauline leaves and fruits in specimens E00000706, HBG510909, and the upper left portion of NY00406258 correspond to S. orthacantha var. orthacantha (Li et al. 2022), rather than to *S. serrata.* Only specimens K001325368, the lower right portion of NY00406258, and US00126980 conform to the original description of *S. serrata.*Pimenov (2017) designated the specimen at K (K001325368) as the lectotype, and the specimens at E, HBG, NY, and US as isolectotypes. In accordance with ICN Article 9.3 (Turland et al. 2025), we accept Pimenov’s (2017) lectotypification and confirm that the lower right portion of NY00406258 and US00126980 represent authentic duplicates and are hereby recognized as isolectotypes.

**2)**Hiroe (1979) classified Sanicula elata var. acaulis as a synonym of *S. serrata.* Upon reviewing the protologue of S. elata var. acaulis, we note that it provides only a brief description, plant 40–50 cm tall, with almost no stem; umbel dense and globose, with very short, angular, winged raylets; calyx lobes triangular, with no type material cited (Franchet 1883). Although Liu (1997) endorsed Hiroe’s treatment, we have not been able to locate any associated specimens or additional corroborating literature. A more detailed assessment of S. elata var. acaulis will be presented elsewhere.

**3)***Sanicula elongata* was described based on two samplings: *P.C. Kuo 1424* and *K.T. Fu 15559*. Fu (in Liou 1979) cited the former collection as the type but did not designate a specific specimen as the holotype. We have traced four specimens referable to the former collections, preserved at KUN, IBK (Fig. 5A–C). All specimens match the original description and are considered syntypes, as Fu (in Liou 1979) did not designate a single type specimen. Although Pimenov (2017) indicated that the specimen *P.C. Kuo 1424* at WUK was the holotype, he did so without examining the specimen, and we were unable to locate a duplicate sheet at WUK. Based on our investigation, the specimen at WUK likely corresponds to *K.T. Fu 15559* (Fig. 5D). In accordance with ICN Article 9.11 (Turland et al. 2025), we designate the specimen *P.C. Kuo 1424* kept at IBK (IBK00159684; Fig. 5A) – which is complete and well preserved – as the lectotype.

**4)**Sanicula tienmuensis var. pauciflora was described based on a single collection, *Y.L. Cao 115*, which was incorrectly attributed to “Y.J. Li” in the protologue. We have identified two specimens of this collection at CDBI, both of which are well-preserved and consistent with the protologue. Since Shan and Pu (1989) did not designate a single type specimen, both specimens qualify as syntypes according to the discussion by McNeill (2014). In accordance with ICN Article 9.17 (Turland et al. 2025), Pimenov’s (2017) reference to a CDBI sheet as the “holotype” is incorrect and should be amended to “lectotype” (McNeill 2014). Consequently, we designate sheet CDBI0172308 (Fig. 6A) as the lectotype and CDBI0172309 (Fig. 6B) as the isolectotype.

#### ﻿2. Sanicula potaninii

Taxon classificationPlantaeApialesApiaceae

Bobrov, Bot. Mater. Gerb. Bot. Inst. Komarova Akad. Nauk S.S.S.R. 13: 168 (1950).

6B92C13F-3746-52D1-9F73-A2CEC5902848

Figs 2, 4, 9, 13, 16 康定变豆菜

= Sanicula serrata var. uncinata R.H. Shan & Constance, Univ. Calif. Publ. Bot. 25(1): 25 (1951), syn. nov. Type: China. Sichuan, Kangding, Cheto Valley, 1923, *R. Cunningham 37* (holotype: E00000048!)

##### Type.

China. • Sichuan, Kangding, 18 May 1893, *G.N. Potanin s.n.* (lectotype: LE01029609!, designated by Vinogradova (2010); isolectotypes: LE01029611!, LE01029608!, LE01029610!, LE01029612!, PE00025857!). Fig. 2

##### Description.

Perennial. Rhizome, short and stout, roots fascicled, fleshy, somewhat fibrous. Stems 2–4, erect, somewhat slender, branched above, 9–45 cm tall. Basal leaves 1–15, long petiolate; petioles 1.5–9(–15) cm long; blade glabrous adaxially and abaxially, 1–4.5 cm long, 2–8 cm wide, rounded-cordate to cordate-pentagonal, tripartite to the base or trifoliate, the median segment cuneate-obovate to rhombic-cuneate, distinctly shallowly trilobed, the lateral segments oblique, usually bilobed shallowly, to the middle or nearly to the base, the margins irregularly crenulate-serrate with attenuated teeth. Cauline leaves few, conspicuous, tripartite nearly to the base, upper stem leaves subsessile or sessile, tripartite, resembling involucrate bract, 0.9–4 cm long, 0.8–5.5 cm wide. Inflorescence usually 3-branched; involucrate bract usually 2, trisect, 0.2–0.4(–2) cm long; rays of the umbels 1–5, 3–24 mm long; involucellate bracteoles 5–7, oblong lanceolate, ca. 0.8 mm long. Umbellules 3–9-flowered, staminate flowers 2–7 per umbellule, pedicels 1.2–1.8 mm long, petals white, fertile flowers 1–3 per umbellule, sessile; calyx teeth triangular shape with a sharp tail, 0.9–1.3 mm long; styles ca. 4.5 mm long, 2–5 times longer than the calyx teeth. Mericarps oblong-ovoid to ovoid, 1.3–2 mm long, 1.5–3 mm broad, with densely purple uncinate spines.

##### Distribution.

*Sanicula potaninii* is restricted to Shaanxi and Sichuan, China (Fig. 18).

##### Habitat.

This species grows on mountain slopes under forest or along ravine streams near rocks at elevations of 2,400–3,400 m above sea level.

##### Phenology.

Flowering from later April to May, fruiting from May to June.

##### Etymology.

The epithet *potaninii* is derived from the Latinized form of the surname Potanin, in honor of the Russian explorer and botanist Grigory Nikolayevich Potanin (1835–1920).

##### Additional specimens examined.

**China. Shaanxi Province**, • Baoji City, Taibaishan, 34°00'37.4"N, 107°49'24.8"E, alt. 2,484 m, 2 June 2022, *H.M. Li, C.F. Song 1200* (NAS); • Baoji City, Taibaishan, 16 June 1957, *Z.L. Wu 30435* (NAS). **Sichuan Province**, • Chongzhou City, Jiguanshan, 30°46'9.75"N, 103°10'11.65"E, alt. 2935 m, 16 June 2016, *W.B. Ju, L. Zhang, D.K. Chen AZH01291* (CDBI); • Jinchuan County, Sayinchanggou, alt. 2,800 m, 26 April 1958, *X. Li 77235* (NAS); • Kangding County, Laoyulin, alt. 2,950 m, 9 May 1980, *Z.Y. Chen, G. Hu, Z.J. Zhao 111918* (SZ); • Kangding County, Longtougou, alt. 2,900 m, 7 May 1981, *Z.J. Zhao, J.B. Shi, D.G. Fan 113916* (CDBI, SZ); • Kangding County, Mount Paoma,30°2'58"N, 101°57'48"E, alt. 2,600–2,649 m, 8 June 2020, *H.M. Li, W. Zhou 1065* (NAS); • Kangding County, Mount Paoma, 30°03'04.7"N, 101°57'45.7"E, alt. 2,574 m, 31 May 2024, *H.M. Li, C.F. Song, J.W. Zhu 1496* (NAS); • Kangding County, Mount Paoma, 30°3'4"N, 101°57'47"E, alt. 2,512 m, 17 May 2021, *H.M. Li, Y.S. Zhang 1163* (NAS); • Kangding County, Mount Paoma, alt. 3,400 m, 23 May 1974, *J.F. Wang 6032* (CDBI, NAS); • Kangding County, Simaqiao, alt. 2,700 m, 12 May 1980, *G. Hu, Z.J. Zhao 111999* (SZ); • Kangding County, Simaqiao Forest Farm, alt. 2,750 m, 22 May 1974, *Y.T. Wu, Q.S. Zhao 11052* (CDBI, SZ, WUK); • Kangding County, Yunlin Township, alt. 2,770 m, 10 May 1980, *Z.Y. Chen, Z.X. Xiong 112113* (CDBI, SZ); • Kangding County, 17 June 1893, *G.N. Potanin s.n.* (LE); • Kangding County, 22 June 1893, *G.N. Potanin s.n.* (LE); • Kangding County, 9 May 1893, *G.N. Potanin s.n.* (LE); • Kangding County, 27 May 1893, *G.N. Potanin s.n.* (LE); • Kangding County, 1897, *R.P. Mussot 160* (P); • Kangding County, *A.C. Young 62* (NAS); • Li County, Jiabigou, alt. 2,700 m, 5 May 1956, *X.S. Zhang 1564* (SZ); • Li County, Jiabigou, 4 May 1956, *X.S. Zhang 1533* (SZ); • Li County, Longxi Township, 10 May 1952, *W.P. Fang, Z. He 12328* (SZ, NAS, PE); • Markang City, Pu’er ma Village, alt. 3,160 m, 24 May 1957, *X.S. Li 70819* (KUN, NAS, PE); • Markang City, Wangjiazhaigou, alt. 3,100 m, 27 June 1957, *Southwest Forestry University Research Lab 84* (NAS); • Markang City, Yingzigou, alt. 2,800 m, 26 May 1957, *X. Li 71176* (NAS); • 1893, *J.A. Soulié s.n.* (P); • 10 June 1952, *Z. He, Z.L. Zhou 12686* (NAS, PE); • *K.L. Chiu 7003* (NAS, PE); • *Tang 1121* (PE).

**Note**. Bobrov (1950) described *S. potaninii* from five samplings collected by *G.N. Potanin* (9, 18, and 27 May 1893; 17 and 22 June 1893), without designating a holotype. We traced 12 specimens corresponding to these samplings at LE and PE, all matching the protologue. Vinogradova (2010) subsequently designated the May 18, 1893 collection as the lectotype, explicitly labelling LE01029609 and listing its duplicates as isolectotypes. We follow this lectotypification.

## Supplementary Material

XML Treatment for Sanicula serrata

XML Treatment for Sanicula potaninii

## ﻿Additional information

### Use of AI

No use of AI was reported.

### Author ORCIDs

Hui-Min Li https://orcid.org/0000-0001-6660-7620

Wei Zhou https://orcid.org/0000-0003-1627-1529

Bao-Cheng Wu https://orcid.org/0009-0006-1719-3517

Xu-Dong Ma https://orcid.org/0009-0006-6303-0006

Jun Wen https://orcid.org/0000-0002-1926-6517

Chun-Feng Song https://orcid.org/0000-0003-2077-0486

### Data availability

All of the data that support the findings of this study are available in the main text or Supplementary Information.

## Supplementary materials

Supplementary material 1 Summary of inflorescence length measurements (cm) for *Sanicula serrata* across nine populations in Hubei, Zhejiang, Shaanxi, and Sichuan Data type xlsx https://binary.pensoft.net/file/1445461This dataset is made available under the Open Database License (http://opendatacommons.org/licenses/odbl/1.0/). The Open Database License (ODbL) is a license agreement intended to allow users to freely share, modify, and use this Dataset while maintaining this same freedom for others, provided that the original source and author(s) are credited.Hui-Min Li, Wei Zhou, Bao-Cheng Wu, Xu-Dong Ma, Jun Wen, Chun-Feng Song
